# Potent and Selective IGF‐IIR‐Recruiting Bifunctional Molecules for Targeted Lysosomal Degradation of Extracellular and Membrane Proteins

**DOI:** 10.1002/advs.202518793

**Published:** 2026-01-28

**Authors:** Yuan Zhao, Yaxian Liao, Pengyun Li, Regina Stasser de Gonzalez, Xuankun Chen, Nicholas S. Nieto, Florence M. Brunel, Nick Cox, Joseph Stock, Matthew McHenry, Guangsen Fu, Penghsuan Huang, Wenxin Wu, Deqin Cai, Lingjun Li, Alexander N. Zaykov, Weiping Tang

**Affiliations:** ^1^ Lachman Institute of Pharmaceutical Development School of Pharmacy University of Wisconsin‐Madison Madison Wisconsin USA; ^2^ Chemical Biology Novo Nordisk US R&D Lexington Massachusetts USA; ^3^ Department of Chemistry University of Wisconsin‐Madison Madison Wisconsin USA

**Keywords:** encodable fusion‐protein, IGF‐IIR, LYTAC, protein degradation

## Abstract

Lysosome targeting chimeras (LYTACs) represent a promising strategy to harness lysosomal degradation for eliminating extracellular and membrane disease‐causing proteins. These bifunctional molecules link a target protein to a lysosome targeting receptor (LTR), forming a ternary complex that drives internalization and degradation. The first generation of LYTAC used cation‐independent mannose‐6‐phosphate receptor (CI‐M6PR), also known as Type II insulin‐like growth factor receptor (IGF‐IIR), as the LTR, with polymeric glycopeptides as the ligands. However, their complex and heterogeneous composition limits therapeutic potential. To improve specificity and efficacy, natural IGF‐II has been explored as an alternative ligand. However, wild‐type IGF‐II activates both Type I insulin‐like growth factor receptor (IGF‐IR) and insulin receptor isoform A (IR‐A), posing off‐target risks. In this study, we engineered a novel IGF‐II mutant (mutIGF‐II) with two mutations (Del1‐7 and Y27L), which confer high affinity for IGF‐IIR while minimizing binding to IGF‐IR and IR‐A. The mutIGF‐II‐based bifunctional degraders significantly enhanced internalization and degradation of both secreted and membrane‐bound proteins. Additionally, we developed a practical all‐protein mutIGF‐II LYTAC by genetically encoding mutIGF‐II into a mammalian expression vector and transfecting it into cancer‐relevant cell lines. The secreted mutIGF‐II‐based PD‐L1 degrader effectively induced PD‐L1 degradation.

## Graphical abstract

Lysosome‐targeting chimeras (LYTACs) enable degradation of extracellular and membrane proteins via lysosomal trafficking. We report a novel IGF‐II mutant (Del1–7, Y27L) that selectively engages IGF‐IIR while avoiding IGF‐IR and IR‐A. mutIGF‐II–based LYTACs enhance target internalization and degradation and support a genetically encodable, all‐protein degrader format.

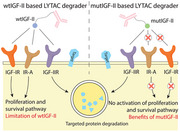

## 1 Introduction

Targeted protein degradation (TPD) technologies exploit the cell's endogenous degradation machinery to selectively induce the degradation of disease‐causing proteins [1, 2, 3, 4, 5, 6, 7]. These technologies typically utilize bifunctional molecules composed of two binding components: one targeting the protein of interest and the other recruiting the degradation machinery to facilitate its destruction. Unlike traditional occupancy‐based therapeutics, which primarily block a protein's function by inhibiting its active site, TPD eliminates the entire protein. This unique mechanism enables TPD to target a broad range of proteins, including those with scaffolding functions or multiple roles within the cell, many of which were previously considered undruggable. Furthermore, TPD often provides sustained signaling suppression for proteins with a slow turnover rate, as the effect of protein elimination can persist even after the degraders have been cleared from the body. Given these and other advantages [8, 9, 10], TPD technologies have emerged as a promising strategy for combating various diseases.

The most advanced TPD strategy employing bifunctional molecules is the proteolysis targeting chimera (PROTAC), which exploits the ubiquitin‐proteasome system, the primary mechanism for intracellular protein degradation [2, 3]. PROTACs operate by recruiting an E3 ubiquitin ligase to facilitate the degradation of specific protein targets. This approach has been extensively investigated in clinical trials for treating cancers and other diseases [4]. Despite their potential, PROTACs are inherently limited to degrading intracellular proteins due to their reliance on the ubiquitin‐proteasome system. This constraint excludes extracellular proteins, which constitute approximately 40% of the human proteome [11]. Lysosomal targeting chimeras (LYTACs) [12] present a complementary protein degradation technology that addresses this gap. LYTAC technology leverages the lysosomal degradation pathway that functions by engaging extracellular proteins [13]. Notably, prior to the recent surge of LYTACs [14, 15, 16, 17, 18, 19, 20], Chugai developed a similar concept known as the Sweeping Antibody [21]. Similar to LYTAC, this platform leverages the recruitment of Fc receptors to mediate the internalization and lysosomal degradation of targeted proteins. This technology represents the first commercialized example of extracellular protein degraders.

The lysosome‐targeting receptor (LTR) used in the first generation of LYTACs was the cation‐independent mannose‐6‐phosphate receptor (CI‐M6PR) [12]. These LYTACs demonstrated internalization and degradation of model soluble targeted protein NeutrAvidin and membrane proteins, such as EGFR. However, the ligands of CI‐M6PR utilized in the first generation of LYTACs were polyglycopeptides containing multiple units of mannose‐6‐phosphonate (M6Pn), synthesized through random copolymerization. This approach resulted in complex mixtures, posing significant challenges for further therapeutic development.

CI‐M6PR is also known as the Type II insulin‐like growth factor receptor (IGF‐IIR). Other IGF‐IIR ligands, such as human IGF‐II, may offer promising alternatives as an LTR binder for LYTAC development. The challenge with native IGF‐II is that it can also bind and activate other receptors, such as the Type I insulin‐like growth factor receptor (IGF‐IR) and insulin receptor (IR), with especially high affinity for the isoform A of IR (IR‐A). Activation of these receptors is associated with mitogenic activities such as cell growth, differentiation, and survival [22, 23, 24]. In contrast, IGF‐II's interaction with IGF‐IIR is not known to produce any downstream signaling. Instead, the primary function of IGF‐IIR in this case is to facilitate ligand clearance by trafficking it to the lysosome for degradation [25]. During our studies, several IGF‐II–based LYTAC strategies were reported; however, these approaches relied on wild‐type IGF‐II or mutants primarily optimized for IGF‐IIR binding [26, 27, 28]. For example, Yang et al. employed a triple mutant IGF‐II in a genetically encoded GELYTAC format to recruit IGF‐IIR for targeted proteins [28]. While these studies demonstrate that IGF‐II‐derived ligands can function as lysosome‐targeting handles, the potential cross‐reactivity of these ligands with IGF‐IR and IR‐A has not been thoroughly evaluated. Consequently, LYTAC degraders incorporating these ligands may carry a risk of mitogenic side effects such as stimulation of cancer cell growth, cardiovascular issues, and growth abnormalities [29]. More recent studies have explored LYTAC designs featuring IGF‐II‐like peptides or engineered IGF‐II mutants with enhanced selectivity for IGF‐IIR over IGF‐IR [30, 31]. However, the interaction of these ligands with IR‐A has not yet been systematically examined.

Several IGF‐II mutants have been reported to exhibit enhanced binding affinity for IGF‐IIR while reducing affinity for IGF‐IR and IR‐A [32, 33, 34, 35]. Among these, the Del1‐7 and Y27L mutations are particularly notable. However, to the best of our knowledge, the combination of these two mutations (Del1‐7 and Y27L) has not been previously reported. In this study, we generated a novel IGF‐II mutant (mutIGF‐II) by incorporating both the Del1‐7 and Y27L mutations. We verified that this mutIGF‐II (Del1‐7/Y27L) maintains high affinity for IGF‐IIR while exhibiting significantly reduced engagement with IGF‐IR and IR‐A. This receptor binding profile suggests that Del1‐7/Y27L can preserve IGF‐IIR‐mediated lysosomal targeting while minimizing IGF‐IR/IR‐A‐driven mitogenic signaling, thereby offering an improved safety profile. By chemically conjugating mutIGF‐II with biotin or antibodies targeting specific proteins, we successfully induced significant internalization and degradation of NA‐650, EGFR, PD‐L1, and Her2. Furthermore, we compared these mutIGF‐II‐based degraders with our previously developed LYTAC degraders containing structurally well‐defined oligomeric M6Pn [36], demonstrating superior efficacy of the mutIGF‐II‐based degraders. Finally, we designed a plasmid encoding a secreted fusion protein based on mutIGF‐II as a PD‐L1 degrader. This construct effectively induced the degradation of PD‐L1 in cancer cells, showcasing the potential of this approach for therapeutic applications.

## 2 Results

### 2.1 mutIGF‐II Selectively Binds to IGF‐IIR with Higher Affinity Than to IGF‐IR and IR‐A

Wild‐type IGF‐II (wtIGF‐II) and mutant IGF‐II (mutIGF‐II) were expressed in *E. coli* and purified using HPLC. Following purification, we evaluated the binding selectivity of mutIGF‐II for IGF‐IIR compared to IGF‐IR and IR‐A using surface plasmon resonance (SPR). Recombinant extracellular domains of human IGF‐IR, IR‐A, and IGF‐IIR (domain 11–13 fragment) were obtained from R&D Systems. Recombinant full‐length extracellular domains of IGF‐IIR or soluble IGF‐IIR (sIGF‐IIR) were kindly provided by Prof. Dahms [37].

**FIGURE 1:**
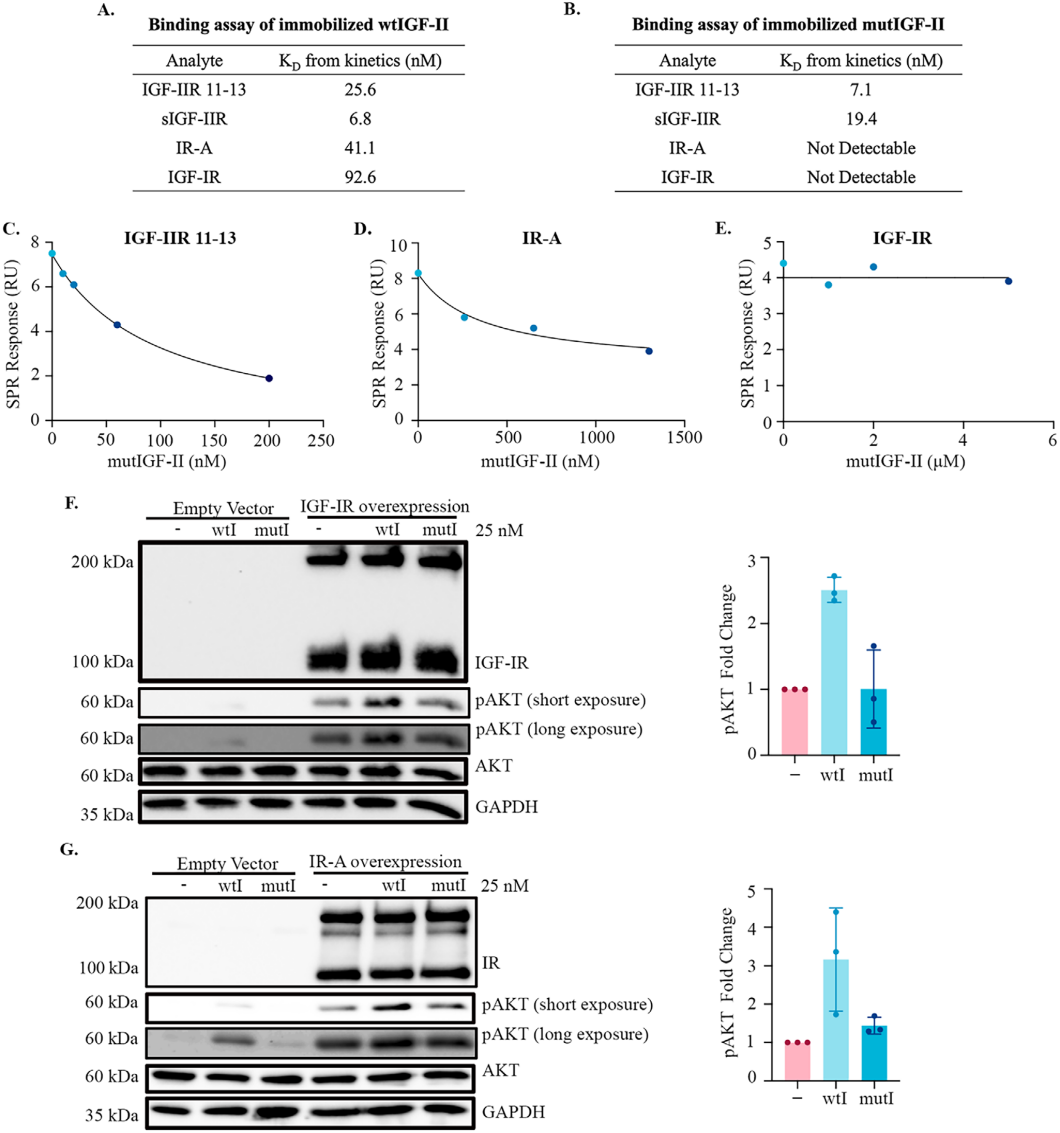
mutIGF‐II selectively binds to IGF‐IIR with higher affinity than to IGF‐IR and IR‐A. (A) Summary table of K_D_ values of wtIGF‐II binding to three receptors determined by SPR. IGF‐IIR 11–13: extracellular domain 11–13 fragment of IGF‐IIR; sIGF‐IIR: full‐length of extracellular domain of IGF‐IIR; IR‐A: recombinant extracellular domain of human IR‐A; IGF‐IR: recombinant extracellular domain of human IGF‐IR). K_D_ values were calculated as the mean values of K_D_ values from replicated kinetic experiments. (B) Summary table of K_D_ values of mutIGF‐II binding to three receptors determined by SPR. (C) Effect of increasing concentrations of mutIGF‐II on the binding of wild‐type IGF‐II to IGF‐IIR 11–13. (D) Effect of increasing concentrations of mutIGF‐II on the binding of wild‐type IGF‐II to IR‐A. (E) Effect of increasing concentrations of mutIGF‐II on the binding of wild‐type IGF‐II to IGF‐IR. ND: not detectable. (F) pAKT activation in HepG2 cells overexpressing IGF‐IR. (G) pAKT activation in HepG2 cells overexpressing IR‐A. (mutI: mutIGF‐II; wtI: wtIGF‐II).

Initially, we characterized the binding properties of wtIGF‐II. Biotinylated wtIGF‐II was immobilized on a streptavidin‐coupled chip, and various concentrations of each receptor were injected to determine binding kinetics (Figure S1). As expected, wtIGF‐II bound to all three receptors (Figure 1A). Subsequently, biotinylated mutIGF‐II was immobilized, confirming its binding to IGF‐IIR with similar affinity to wtIGF‐II (Figure 1B). In contrast, binding of immobilized mutIGF‐II to IGF‐IR or IR‐A was undetectable by SPR (Figure S2).

To further investigate the binding selectivity of mutIGF‐II, we performed a competition assay using immobilized wtIGF‐II. Receptors were pre‐incubated with varying concentrations of mutIGF‐II prior to injection. This allowed us to evaluate the ability of mutIGF‐II to compete with wtIGF‐II for receptor binding. For IGF‐IIR (20 nM), a 3‐fold molar excess of mutIGF‐II (60 nM) resulted in approximately 50% inhibition of wtIGF‐II binding, while a 10‐fold molar excess of mutIGF‐II (200 nM) yielded ≈80% inhibition (Figure 1C; Figure S3). Conversely, 50% inhibition of binding to IR‐A (13 nM) required a 100‐fold molar excess of mutIGF‐II (Figure 1D; Figure S4). Notably, IGF‐IR binding (25 nM) was refractory to inhibition even in the presence of a 200‐fold molar excess of mutIGF‐II (5 µm) (Figure 1E; Figure S5). An additional competition assay was also performed, where a 5‐fold molar excess of mutIGF‐II was pre‐incubated with the receptors before injection onto the immobilized wtIGF‐II surface. Only the IGF‐IIR/wtIGF‐II interaction was abolished, while binding to IGF‐IR and IR‐A remained unaffected (Figure S6). These results clearly indicate that the designed mutIGF‐II selectively binds to IGF‐IIR with higher affinity than to IGF‐IR and IR‐A.

To corroborate these biophysical findings in a cellular context, we assessed receptor activation via AKT phosphorylation (pAKT) levels. Ligand engagement of IGF‐IR or IR‐A activates downstream AKT phosphorylation (pAKT), so pAKT levels provide a readout of receptor activation. Using HepG2 cells, which were confirmed to express endogenous IGF‐IIR, IGF‐IR, and IR (Figure S7), we individually overexpressed IGF‐IR or IR‐A to isolate their specific signaling contributions. The cells were then treated with either wtIGF‐II or mutIGF‐II, followed by measurement of pAKT levels. While wtIGF‐II treatment elicited a significant increase in pAKT levels in both overexpression models, mutIGF‐II exhibited negligible signaling activity, even under conditions of receptor overexpression (Figure 1F,G). These results indicate that mutIGF‐II has markedly reduced functional engagement of IGF‐IR and IR‐A compared with wtIGF‐II.

### 2.2 Biotinylated mutIGF‐II Induces Internalization of NeutrAvidin‐650 (NA‐650)

**FIGURE 2:**
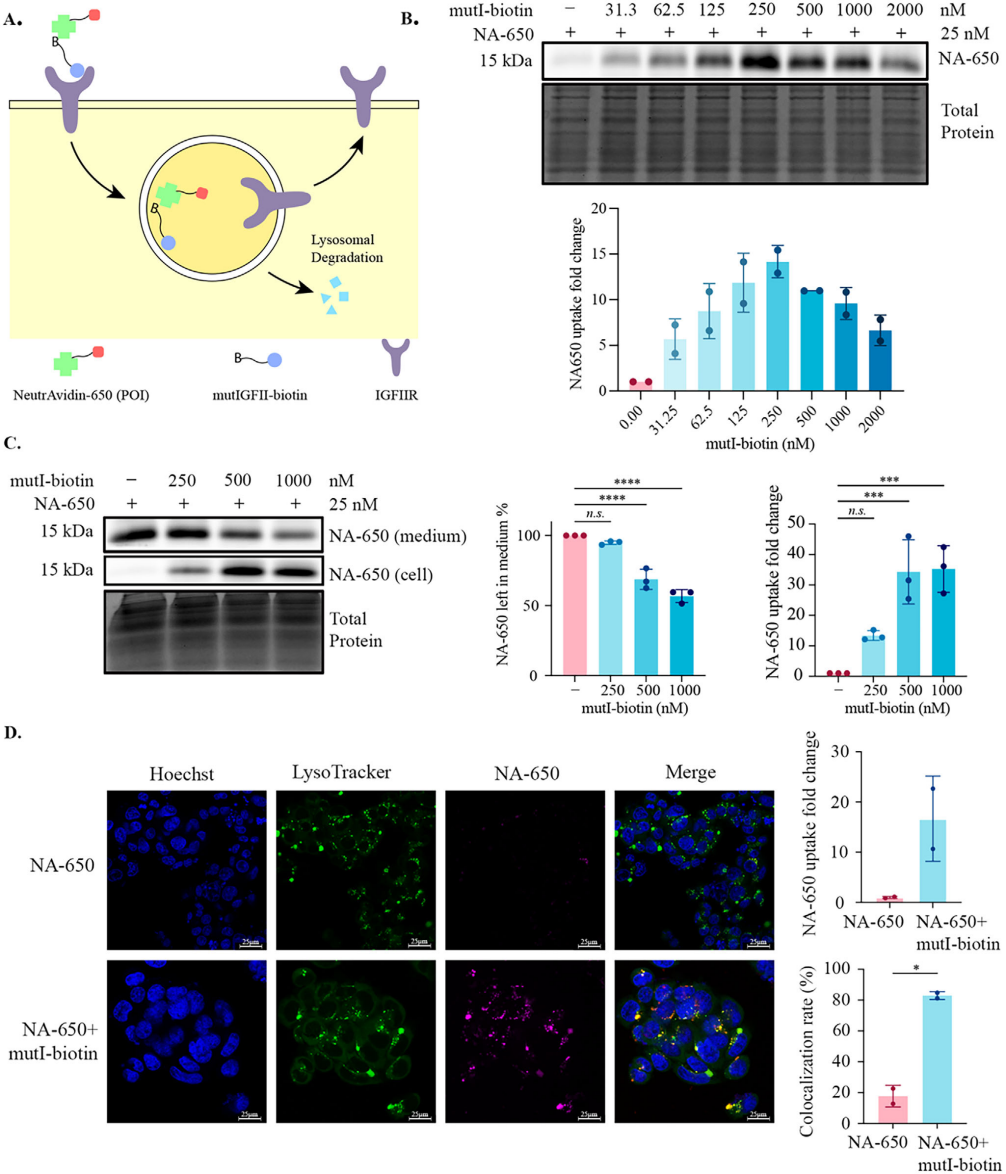
Biotinylated mutIGF‐II induces internalization and lysosomal trafficking of NA‐650. (A) Schematic representation of NA‐650 uptake induced by biotinylated mutIGF‐II. (B) Dose‐dependent NA‐650 uptake induced by biotinylated mutIGF‐II in HepG2 cells after 24 h treatment. (C) Significant NA‐650 depletion from serum‐free medium induced by biotinylated mutIGF‐II following 24 h incubation with HepG2 cells. (D) Confocal microscopy images showing colocalization of internalized NA‐650 and LysoTracker in HepG2 cells after 24 h treatment. Scale bar: 25 µm. Data are presented as mean ± SD. The statistical significance was determined using one‐way ANOVA and an unpaired two‐tailed t test, *p < 0.05, **p < 0.01, ***p < 0.001, ****p < 0.0001, ns: not significant. (mutI: mutIGF‐II).

After confirming the selectivity of mutIGF‐II, we developed LYTAC degraders targeting a model protein, fluorescent NeutrAvidin (NA‐650). The mutIGF‐II‐based degrader was generated by conjugating mutIGF‐II to NHS‐PEG_12_‐biotin, enabling simultaneous binding to NA‐650 and selective targeting of IGF‐IIR to induce NA‐650 internalization (Figure 2A). Internalization efficacy was quantified by measuring intracellular fluorescence intensity. Biotinylated mutIGF‐II induced the internalization of NA‐650 in a dose‐dependent manner in HepG2 cells (Figure 2B). Notably, NA‐650 uptake showed a bell‐shaped dependence on biotinylated mutIGF‐II concentration, with a decrease at the highest dose, which is consistent with the high‐dose ‘hook effect’ commonly observed for bifunctional degrader systems [38]. This phenomenon is typically attributed to the saturation of individual binding sites at high ligand concentrations. Efficient uptake requires that a single bifunctional ligand simultaneously engage the receptor and the cargo to form a ternary complex. At low to intermediate ligand concentrations, this bridging is favored, and the amount of ternary complex increases. At very high ligand concentrations, excess ligand favors the formation of binary complexes (ligand‐receptor or ligand‐cargo) over the productive ternary complexes required for internalization, which decreases the overall uptake. Corroborating the internalization data, we also observed significant depletion of NA‐650 from serum‐free medium following biotinylated mutIGF‐II treatment (Figure 2C). Additionally, confocal imaging confirmed that biotinylated mutIGF‐II induced lysosomal trafficking of NA‐650. After 24 h of treatment with NA‐650 and biotinylated mutIGF‐II, HepG2 cells were stained with the fluorescent dye LysoTracker. Clear colocalization of NA‐650 with lysosomes was observed (Figure 2D). For comparison, a wtIGF‐II‐based NA‐650 degrader was also generated, revealing similar internalization efficiency to that of mutIGF‐II (Figure S8).

**FIGURE 3:**
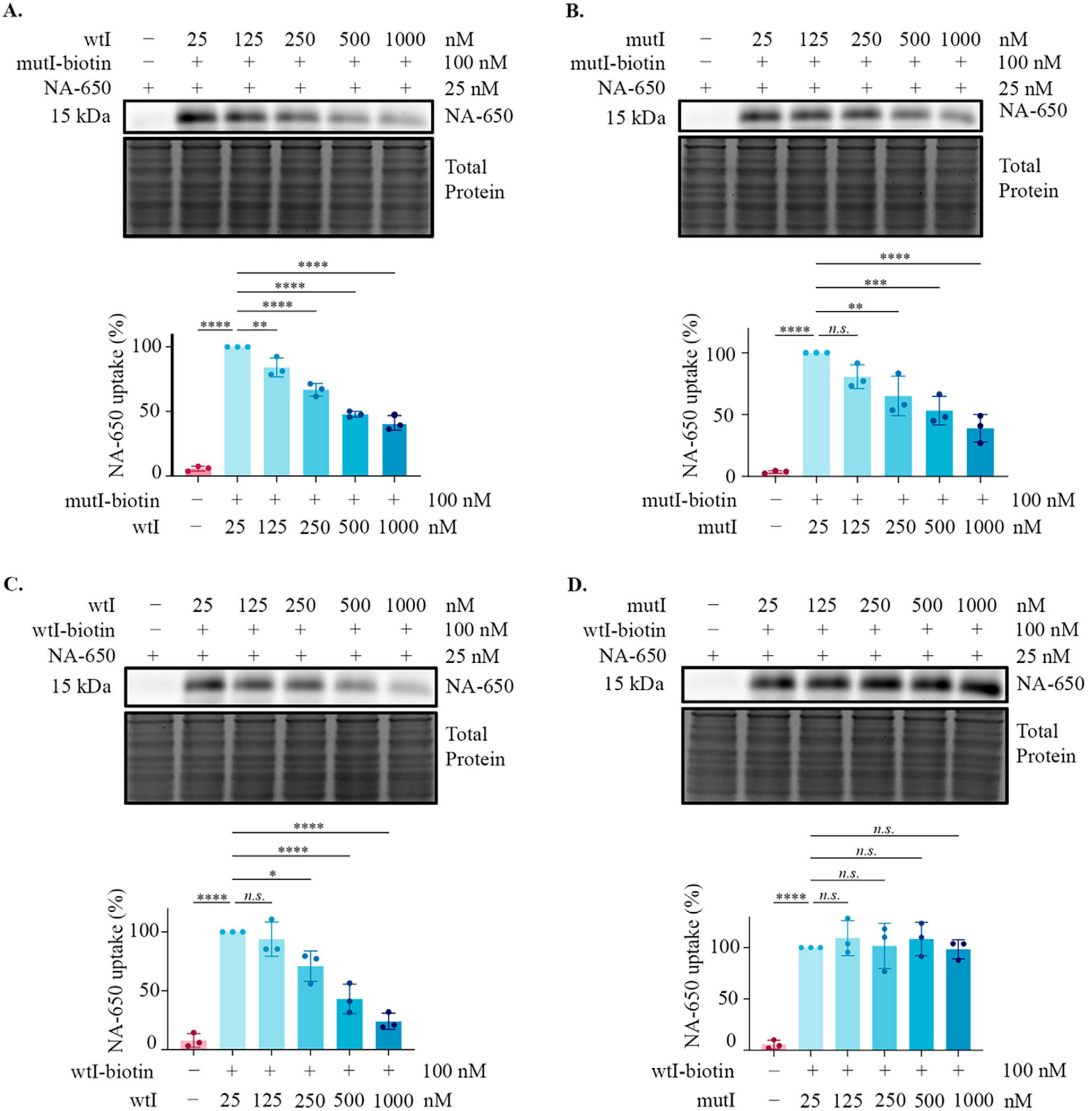
mutIGF‐II selectively inhibits internalization induced by mutIGF‐II‐biotin but not wtIGF‐II‐biotin. (A, B) Inhibition of NA‐650 uptake induced by biotinylated mutIGF‐II in HepG2 cells by excess (A) wtIGF‐II or (B) mutIGF‐II for 24 h. (C) Inhibition of NA‐650 uptake induced by biotinylated wtIGF‐II by excess wtIGF‐II in HepG2 cells. (D) Lack of inhibition of NA‐650 uptake induced by biotinylated wtIGF‐II in the presence of excess mutIGF‐II in HepG2 cells. Data are presented as mean ± SD. The statistical significance was assessed using one‐way ANOVA, ^*^ *p* < 0.05, ^**^ *p* < 0.01, ^***^ *p* < 0.001, ^****^ *p* < 0.0001, ns: not significant. (wtI: wtIGF‐II).

Given that mutIGF‐II selectively binds IGF‐IIR, whereas wtIGF‐II binds to all three receptors, we designed a cell‐based competition assay to confirm this selectivity in a cellular environment. HepG2 cells have been validated to express all three receptors: IGF‐IIR, IGF‐IR, and IR (Figure S7) [39, 40, 41]. Cells were incubated with wtIGF‐II or mutIGF‐II as competitors, along with biotinylated wtIGF‐II or biotinylated mutIGF‐II as degraders of NA‐650. Excess wtIGF‐II effectively abolished uptake induced by both biotinylated wtIGF‐II and mutIGF‐II. In contrast, excess mutIGF‐II selectively blocked the uptake induced by biotinylated mutIGF‐II, without affecting uptake induced by wtIGF‐II (Figure 3).

These findings indicate that wtIGF‐II can internalize via alternative receptors that are not recognized by mutIGF‐II. The data from our cellular assays are consistent with the biochemical binding studies and cellular pAKT activation studies, further validating the designed mutIGF‐II as a selective and potent ligand for IGF‐IIR. This selectivity highlights its potential as a targeted tool for receptor‐specific LYTAC degrader applications, offering a promising approach for receptor‐selective therapeutic interventions.

### 2.3 mutIGF‐II‐Based Degraders Induced Degradation of Membrane Proteins

**FIGURE 4:**
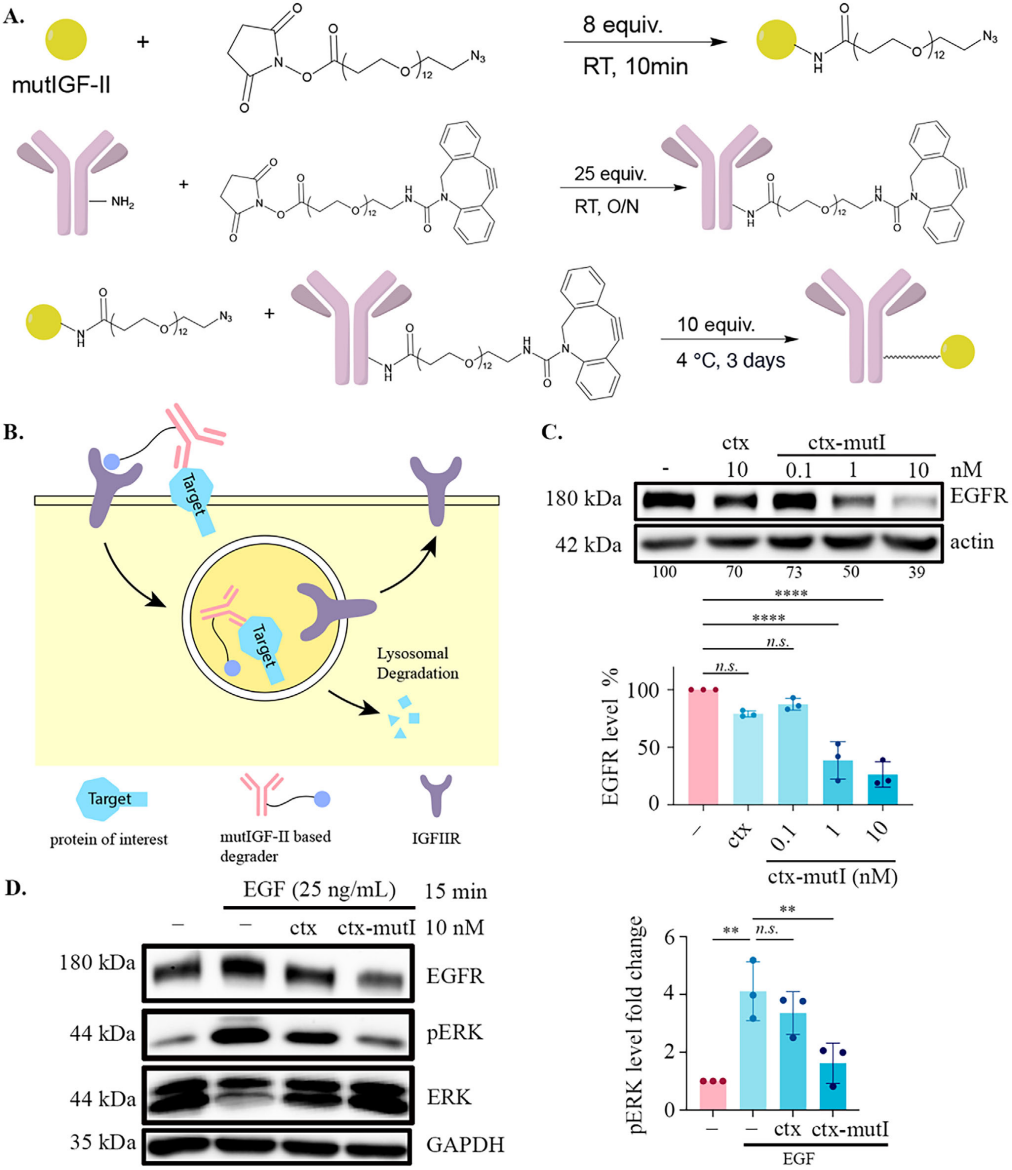
mutIGF‐II‐based degrader induces targeted degradation of EGFR and suppresses downstream signaling. (A) Schematic illustration of the chemical labeling method to generate mutIGF‐II‐based LYTAC degrader. (B) Illustration of membrane protein degradation induced by mutIGF‐II‐based LYTAC degrader. (C) EGFR degradation in Huh7 cells following treatment with the mutIGF‐II‐based degrader for 24 h. (D) Suppression of downstream ERK signaling in HepG2 cells. Western blot analysis showing reduced pERK levels following 24 h degrader treatment and subsequent 15 min EGF stimulation. Data are presented as mean ± SD. The statistical significance was assessed using one‐way ANOVA, ^*^ *p* < 0.05, ^**^ *p* < 0.01, ^***^ *p* < 0.001, ^****^ *p* < 0.0001, ns: not significant.

To extend beyond targeting the model protein NA‐650, we evaluated the degradation of therapeutically relevant membrane proteins (Figure 4B). To achieve this, we developed degraders using a chemical labeling method. First, mutIGF‐II was modified with NHS‐PEG_12_‐azide. Given that mutIGF‐II contains only one lysine residue, the NHS ester can react with two potential sites on mutIGF‐II: the lysine residue and the N‐terminal amine. Because blocking the N‐terminus may influence the activity of mutIGF‐II, we aimed to site‐specifically label the lysine residue of mutIGF‐II. By exploiting the differential reactivity of lysine and the N‐terminal amine with NHS esters, we achieved selective lysine modification by conducting the reaction at pH 11.0 in sodium carbonate buffer.

To characterize the labeling sites of NHS‐PEG_12_‐azide on mutIGF‐II, the conjugates were directly infused and analyzed using a top‐down approach on the Bruker timsToF fleX. A mass shift of 626 Da confirmed the attachment of a single NHS ester on mutIGF‐II (Figure S9). To further identify the specific labeling site, native top‐down MS/MS characterization was performed. For the C‐terminal sequence of mutIGF‐II, *y‐*ions were analyzed to determine whether the NHS ester modification occurred at a lysine (K) residue. The y_2‐7_^1+^ ion series corresponding to the ATPAKSE fragment was detected in mutIGF‐II MS/MS spectra.

However, in mutIGF‐II‐PEG_12_‐azide spectra, only the y_2_^1+^ ion corresponding to the SE di‐peptide fragment was observed, indicating that the modification occurred at the lysine (K) residue within the C‐terminal sequence, as no further fragmentation beyond K was detected (Figure S10). Additionally, *b‐*ions were examined to assess potential modification at the N‐terminus. The b_2_ ion series corresponding to the LC fragment was present in both mutIGF‐II and mutIGF‐II‐PEG_12_‐azide spectra, indicating that the N‐terminal region remained unmodified (Figure S11). These findings confirmed successful site‐specific conjugation of NHS‐PEG_12_‐azide to the lysine residue within mutIGF‐II.

In parallel, a target‐specific antibody was labeled with DBCO via reaction with NHS‐PEG_12_‐DBCO. In the second step, the DBCO‐labeled antibody and azide‐modified mutIGF‐II were conjugated through a copper‐free click reaction (Figure 4A). The average number of mutIGF‐II molecules conjugated to each antibody was determined using MALDI‐TOF MS.

By conjugating cetuximab, an EGFR‐targeting antibody, with mutIGF‐II, we created a degrader targeting EGFR and tested its ability to degrade EGFR. Each cetuximab antibody was labeled with an average of ≈2 mutIGF‐II moieties (Figures S15 and S16). The mutIGF‐II‐based degrader induced EGFR degradation in a dose‐dependent manner in Huh7 cells. The treatment with 1 nM of the degrader for 24 h induced approximately 60% degradation of EGFR, while 10 nM of the degrader induced 70–80% degradation (Figure 4C). To determine whether the observed EGFR degradation translates into functional signaling suppression, we assessed downstream ERK phosphorylation (pERK) levels. Cells were treated with cetuximab or the mutIGF‐II‐based degrader (ctx‐mutIGF‐II), stimulated with EGF, and pERK levels were analyzed by Western blot. As shown in Figure 4D, ctx‐mutIGF‐II treatment resulted in markedly lower EGF‐induced pERK levels than cetuximab, indicating more effective inhibition of the EGFR–MAPK pathway that promotes cell proliferation.

**FIGURE 5:**
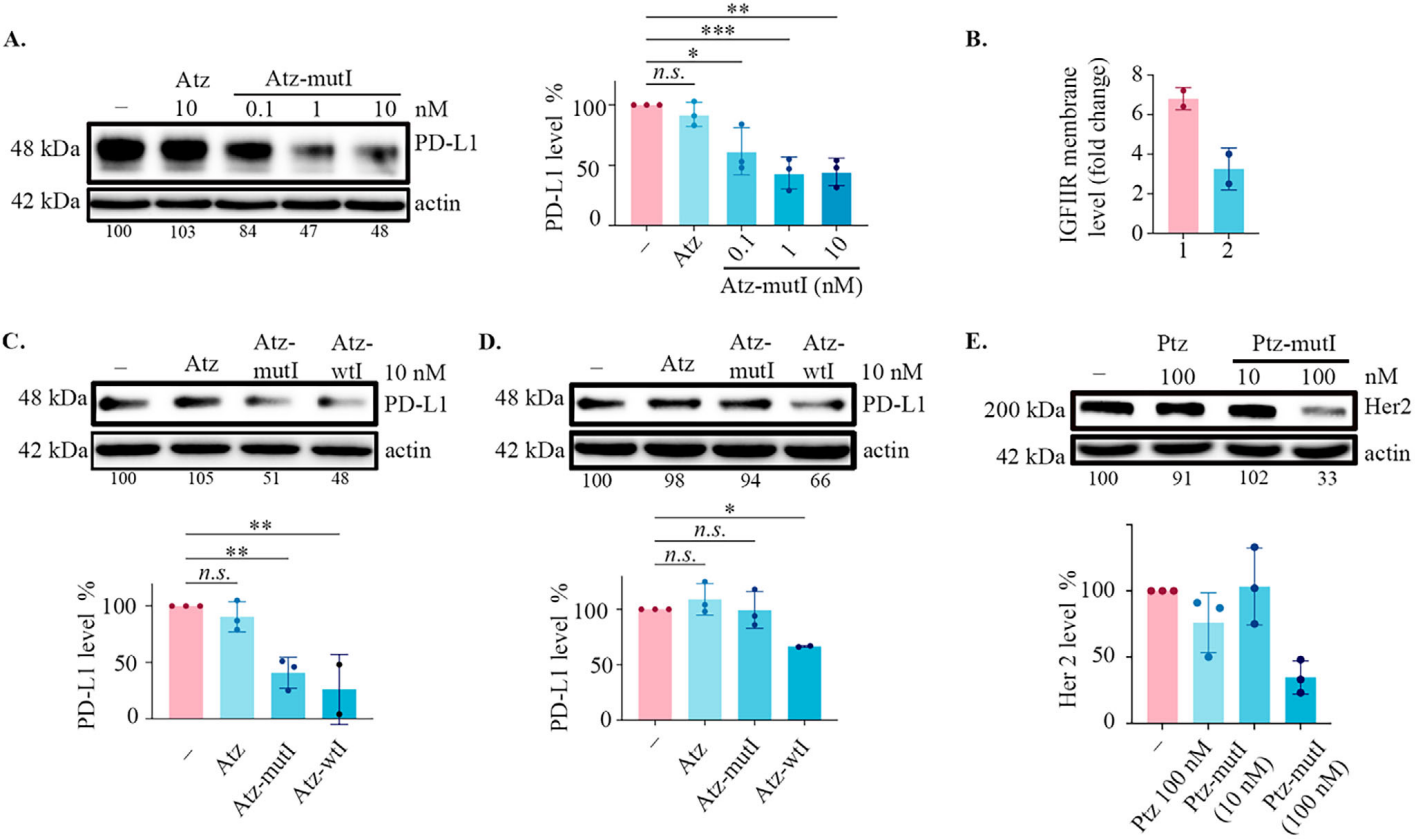
mutIGF‐II‐based degraders induce significant degradation of PD‐L1 and Her2. (A) Degradation of PD‐L1 in MDA‐MB‐231 cells following treatment with the mutIGF‐II‐based degrader for 48 h. (B) Flow cytometry analysis of membrane IGF‐IIR levels. 1: wild‐type Huh7 cells. 2: IGF‐IIR knockdown Huh7 cells. (C) In interferon‐gamma primed wild‐type Huh7 cells, PD‐L1 degradation following treatment with mutIGF‐II‐ or wtIGF‐II‐based degrader for 24 h. (D) Abolishment of PD‐L1 degradation induced by the mutIGF‐II‐based degrader in interferon‐gamma primed IGF‐IIR knockdown Huh7 cells, whereas the wtIGF‐II‐based degrader retains partial activity. (E) Degradation of Her2 in SK‐BR‐3 cells following treatment with mutIGF‐II‐based degrader for 48 h. Data are presented as mean ± SD. The statistical significance was assessed using one‐way ANOVA, ^*^ *p* < 0.05, ^**^ *p* < 0.01, ^***^ *p* < 0.001, ^****^ *p* < 0.0001, ns: not significant.

Furthermore, we generated a degrader targeting PD‐L1 by conjugating mutIGF‐II with atezolizumab, a PD‐L1 targeting antibody. As with the EGFR degrader, an average of 2 mutIGF‐II molecules were conjugated to each atezolizumab antibody (Figures S15 and S17). The PD‐L1 degrader also induced PD‐L1 degradation in a dose‐dependent manner in MDA‐MB‐231 cells. Treatment with 1 nM of the mutIGF‐II‐based degrader induced 60–70% PD‐L1 degradation (Figure 5A). Finally, to assess the impact of mutIGF‐II versus wtIGF‐II, we also developed LYTAC degraders of EGFR and PD‐L1 based on wtIGF‐II and found that mutIGF‐II‐ and wtIGF‐II‐based degraders exhibited similar degradation efficiencies (Figure S12).

To elucidate the role of IGF‐IIR in PD‐L1 degradation mediated by mutIGF‐II‐based degraders, we employed sgRNA‐mediated gene editing to downregulate IGF‐IIR expression in Huh7 cells. Flow cytometry analysis revealed a ∼50% reduction in membrane IGF‐IIR levels after transfection (Figure 5B). Subsequently, we quantified PD‐L1 degradation following treatment with 10 nM of either the mutIGF‐II‐ or wtIGF‐II‐based degrader for 24 h. In wild‐type Huh7 cells, both mutIGF‐II‐ and wtIGF‐II‐based degraders resulted in a significant reduction in PD‐L1 levels (Figure 5C). However, in Huh7 cells with IGF‐IIR knockdown, PD‐L1 degradation induced by the mutIGF‐II‐based degrader was completely abolished, while the wtIGF‐II‐based degrader still induced about 40% PD‐L1 degradation (Figure 5D). These results demonstrate that PD‐L1 degradation mediated by mutIGF‐II‐based degraders is dependent on the presence of IGF‐IIR.

Lastly, using the same labeling method described above (Figure 4A), we also generated a Her2‐targeting degrader by conjugating mutIGF‐II to the anti‐Her2 antibody pertuzumab. On average, around 2 mutIGF‐II molecules were conjugated to each pertuzumab antibody (Figure S18). A 48 h treatment with the degrader resulted in a ∼60–70% degradation of Her2 in SK‐BR‐3 cells (Figure 5E).

### 2.4 mutIGF‐II‐Based LYTAC Degraders Exhibit Greater Potency than Glycopeptide‐Based LYTAC Degraders

The mutIGF‐II‐based LYTAC degraders demonstrated significantly greater potency than LYTAC degraders derived from our previously developed glycopeptide ligands. IGF‐IIR has 15 extracellular repeat domains containing different ligand‐binding sites. IGF‐II binds specifically to domain 11, while the carbohydrate ligands bind to domains 1–3 and 7–9 [42, 43, 44]. Our group previously reported structurally well‐defined oligomeric glycopeptides containing six units of the M6P motif (M6Pn) and developed LYTAC degraders based on these glycopeptide ligands [36] (Figure S13). In this study, we compared these two types of LYTAC degraders, both of which recruit the same LTR (IGF‐IIR or CI‐M6PR).

**FIGURE 6:**
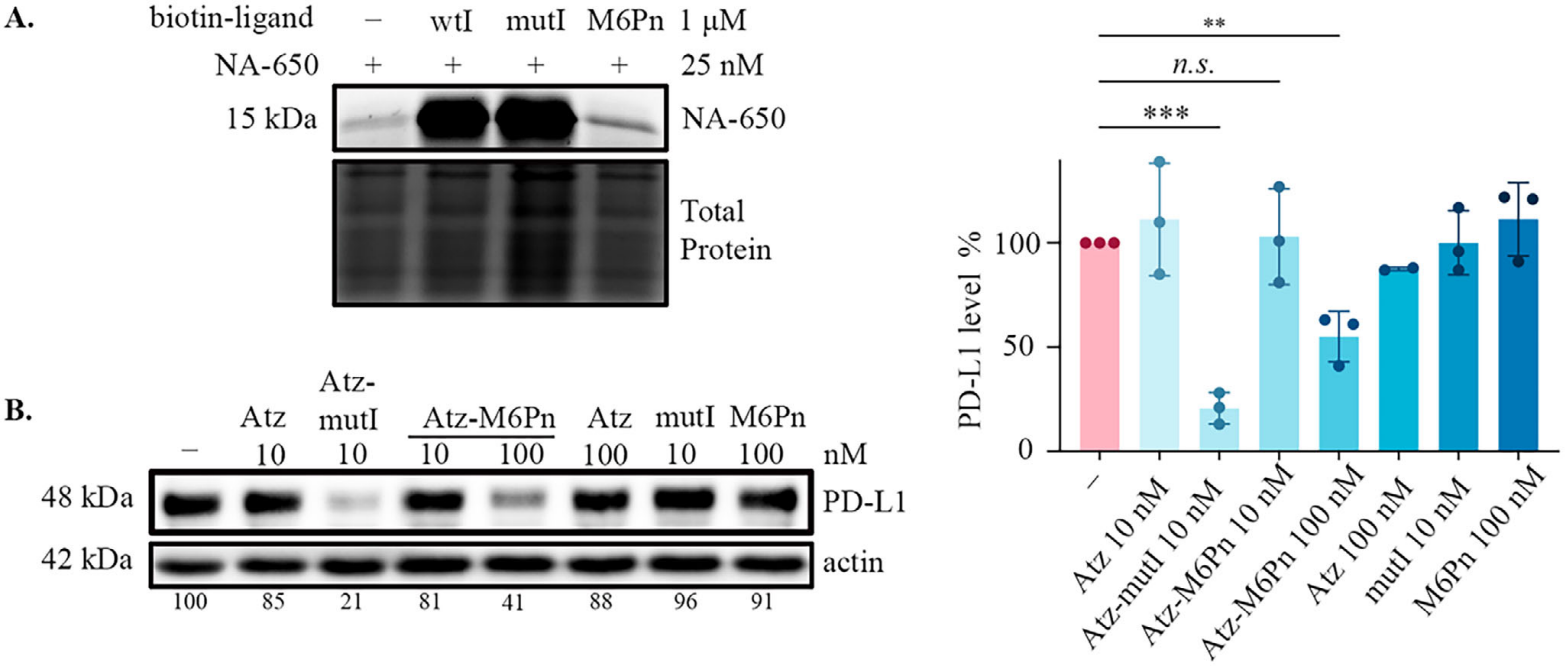
mutIGF‐II‐based degraders exhibit superior potency compared to M6Pn‐based degraders. (A) Comparison of NA‐650 internalization efficiency induced by biotinylated mutIGF‐II versus biotinylated M6Pn. (B) Comparison of PD‐L1 degradation in U87 cells treated with mutIGF‐II‐ versus M6Pn‐based degraders for 24 h, demonstrating the significantly higher potency of the mutIGF‐II‐based degrader. Data are presented as mean ± SD. The statistical significance was assessed using one‐way ANOVA, ^**^ *p* < 0.01, ^***^ *p* < 0.001, ns: not significant.

We first characterized the binding profile of M6Pn. Because M6Pn binds to a domain of IGF‐IIR distinct from the mutIGF‐II binding site, extracellular domains 1–9 were used for the SPR measurements. M6Pn exhibited significantly lower affinity for IGF‐IIR (K_D_ ≈300 nM) compared to mutIGF‐II (K_D_ ≈7.1 nM), while showing no detectable binding to IGF‐IR or IR‐A (Figure S14). We next investigated whether this difference in affinity translates into corresponding differences in cellular uptake and degradation efficiency. Using the NA‐650 model, the mutIGF‐II‐based degrader demonstrated markedly superior uptake compared to the glycopeptide‐based degrader (mutIGF‐II vs M6Pn, Figure 6A). In addition, we conjugated M6Pn to atezolizumab to generate a PD‐L1 degrader using the same labeling method (Figure S17). We then compared the PD‐L1 degradation efficiency of the mutIGF‐II‐based degrader with that of the glycopeptide‐based degrader in U87 cells. Treatment of the cells with 10 nM of the mutIGF‐II‐based degrader induced 80%–90% PD‐L1 degradation in U87 cells, whereas 10 nM of the glycopeptide‐based degrader had no effect (mutIGF‐II vs M6Pn, Figure 6B). Even at a concentration of 100 nM, the glycopeptide‐based degrader only induced about 40%–50% PD‐L1 degradation (Figure 6B).

Collectively, these results demonstrate that while M6Pn shares the safety benefit of avoiding IGF‐IR and IR‐A engagement, its relatively modest affinity for IGF‐IIR limits its potency. Conversely, mutIGF‐II combines high selectivity with nanomolar affinity, resulting in superior cellular uptake and more efficient target protein degradation.

### 2.5 A Genetically Encodable mutIGF‐II Fusion Protein Induces Efficient PD‐L1 Degradation

**FIGURE 7:**
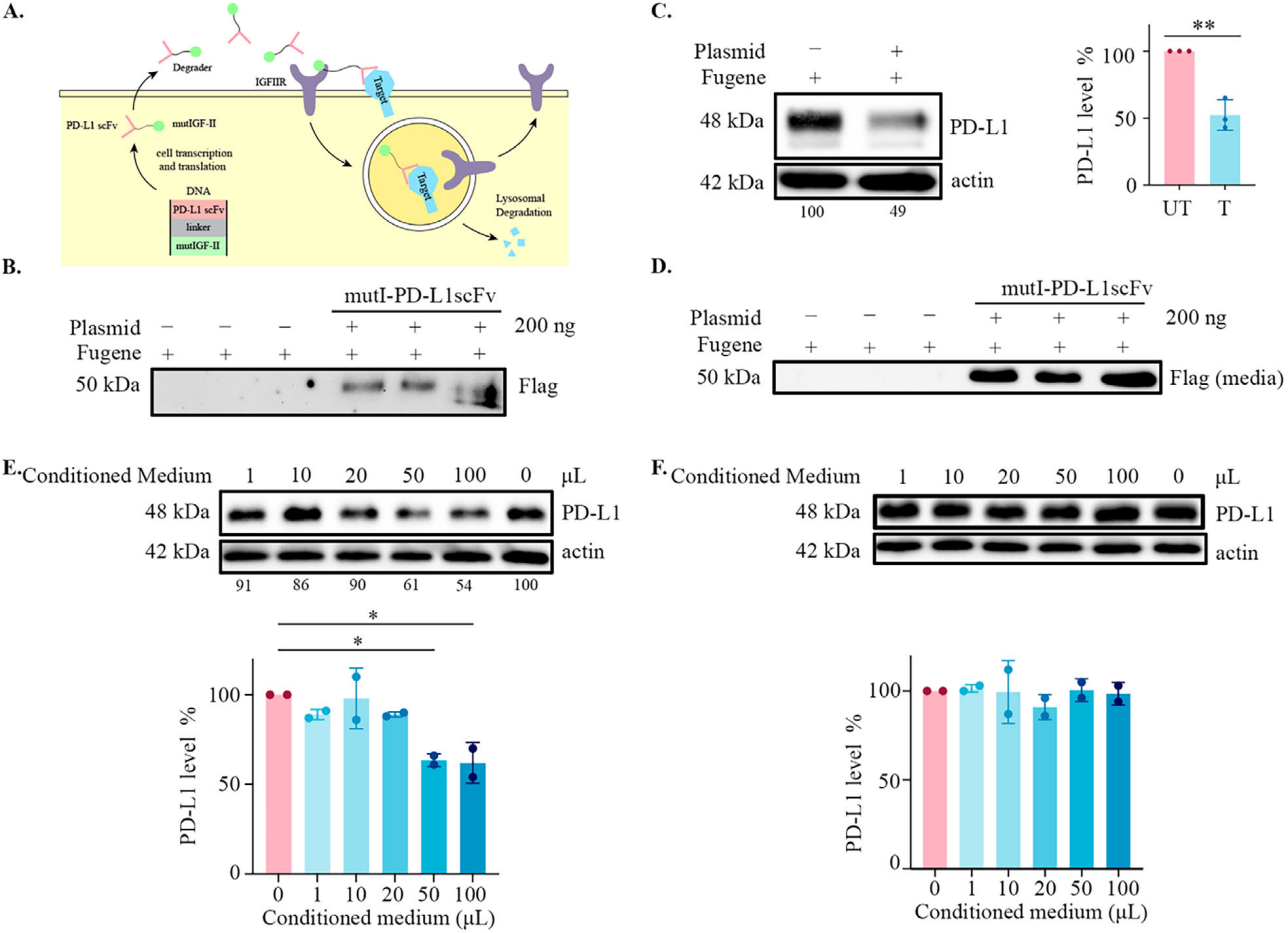
A Genetically encodable fusion protein degrader induces PD‐L1 degradation. (A) Schematic representation of PD‐L1 degradation induced by the encodable fusion protein degrader. (B) Secretion of FLAG‐tagged PD‐L1 degrader from transfected U87 cells following 24 h transfection and a subsequent 48 h incubation. (C) PD‐L1 degradation in transfected U87 cells. UT: untransfected U87 cells. T: transfected U87 cells. (D) Secretion of the FLAG‐tagged degrader from transfected Huh7 cells following 24 h of transfection and a subsequent 48 h incubation. (E) Dose‐dependent PD‐L1 degradation in interferon‐gamma‐primed wild‐type Huh7 cells treated with conditioned medium containing the fusion protein degrader for 24 h. (F) Absence of PD‐L1 degradation in interferon‐gamma‐primed IGF‐IIR knockdown Huh7 cells treated with the same conditioned medium, confirming IGF‐IIR dependency. Data are presented as mean ± SD. The statistical significance was assessed using one‐way ANOVA and an unpaired two‐tailed *t* test, ^*^ *p* < 0.05, ^**^ *p* < 0.01.

In addition to developing LYTAC degraders using the chemical labeling method, we designed an encodable fusion protein degrader by genetically fusing a single‐chain variable fragment (scFv) targeting PD‐L1 to mutIGF‐II. A FLAG tag was incorporated into the fusion protein to facilitate detection and purification (Figure 7A).

To evaluate the expression and secretion of the fusion protein degrader, U87 cells were transfected with the corresponding plasmid. Following 24 h of transfection and a further 48 h incubation after medium replacement, an anti‐FLAG signal was detected in the culture medium of transfected U87 cells, indicating successful secretion of the fusion protein degrader (Figure 7B). The secreted fusion protein degrader induced PD‐L1 degradation in transfected U87 cells (Figure 7C). To explore whether the plasmid can be used in different cell lines, we also transfected Huh7 cells and confirmed that the fusion protein degrader was efficiently secreted from Huh7 cells (Figure 7D).

We next investigated whether the secreted fusion protein degrader present in the conditioned medium could induce PD‐L1 degradation in non‐transfected cells. After 24 h of interferon‐gamma induction, wild‐type Huh7 cells or IGF‐IIR knockdown Huh7 cells were treated with different volumes of conditioned medium collected from transfected Huh7 cells. Dose‐dependent PD‐L1 degradation was observed in wild‐type Huh7 cells, while no PD‐L1 degradation was detected in IGF‐IIR knockdown Huh7 cells (Figure 7E,F).

### 2.6 mutIGF‐II‐biotin Facilitates Deep Penetration and Internalization of NA‐650 in 3D Tumor Spheroids

**FIGURE 8:**
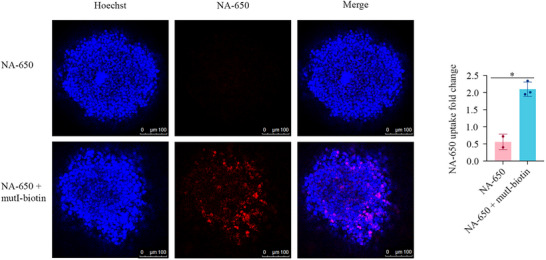
Internalization of NA‐650 in Huh7 3D tumor spheroids induced by mutI‐biotin. Scale bar: 100 µm. Data are presented as mean ± SD. The statistical significance was assessed using an unpaired two‐tailed *t* test, ^*^ *p* < 0.05.

To further validate the therapeutic potential of our approach, we assessed the internalization efficacy in Huh7 3D tumor spheroids. Multicellular tumor spheroids provide a 3D architecture with diffusion barriers and tight cell‐cell contacts that better mimic key features of solid tumors than 2D monolayers. In the absence of mutIGF‐II‐biotin, NA‐650 exhibited limited accumulation within the spheroids. In contrast, co‐treatment with mutIGF‐II‐biotin facilitated a marked increase in the NA‐650 signal throughout the spheroid interior (Figure 8). These results indicate that mutIGF‐II can actively drive cargo penetration into dense 3D tumor spheroids rather than being restricted to the outer cell layers. This suggests that mutIGF‐II‐based LYTACs have the potential to penetrate 3D tumor tissue and access targets located deep within the tumor, representing a crucial step toward achieving efficient protein degradation in solid tumors.

## 3 Conclusion

In summary, we successfully developed a novel mutant IGF‐II (mutIGF‐II) by introducing two mutations, Del1‐7 and Y27L, to increase the selectivity for IGF‐IIR over IGF‐IR and IR‐A. Both SPR assays and cell‐based experiments confirmed that mutIGF‐II selectively binds to IGF‐IIR with significantly higher affinity than to IGF‐IR and IR‐A. This selective binding minimizes the risk of side effects associated with activating the IGF‐IR and IR‐A pathways, which are known to promote cell growth and survival, thereby offering a safer therapeutic window for targeted protein degradation.

We demonstrated that mutIGF‐II‐based LYTAC degraders effectively induce the internalization and degradation of both NA‐650, a model protein target, and therapeutically relevant membrane proteins, such as EGFR, PD‐L1, and Her2. Notably, in 3D tumor spheroids, mutIGF‐II–biotin also increased NA‐650 accumulation throughout the spheroid interior, indicating that mutIGF‐II‐based constructs can penetrate dense tumor‐like structures, which is an important prerequisite for efficient target degradation in solid tumors. Furthermore, mutIGF‐II exhibited significantly superior potency compared to M6Pn‐based glycopeptide degraders, attributed to its higher receptor affinity.

Taken together, this work establishes Del1‐7/Y27L as a receptor‐selective IGF‐II ligand with a favorable IGF‐IIR/IGF‐IR/IR‐A binding and signaling profile. Our study distinguishes itself from previous IGF‐II‐based LYTAC platforms (e.g., GELYTAC and LYTAF) by providing a comprehensive characterization that explicitly defines the selectivity profile for both IGF‐IR and IR‐A, a critical safety parameter that has not been thoroughly explored in this class of degraders. These findings position mutIGF‐II as a promising candidate for next‐generation lysosomal‐targeting chimeras. While our data highlight the favorable profile of Del1‐7/Y27L, future studies that directly compare Del1‐7/Y27L with other IGF‐II mutants used in GELYTAC and LYTAF under matched targets and cell settings will be informative for further optimizing IGF‐II‐based LYTAC design.

## 4 Experimental Section

### 4.1 Expression of wtIGF‐II and mutIGF‐II

Rosetta 2(DE3)pLys competent cells were transformed with the desired plasmid and plated onto LB agar containing ampicillin (100 µg/mL) and chloramphenicol (17 µg/mL). Following overnight incubation at 37°C, colonies were selected and used to inoculate a 100 mL starter culture of LB medium supplemented with ampicillin (100 µg/mL) and chloramphenicol (17 µg/mL). This culture was grown overnight at 37°C with shaking. The starter culture was then used to inoculate 1 L of LB medium containing the same antibiotic concentrations. The culture was incubated with shaking at 37°C until an optical density at 600 nm of 0.8 was reached. The temperature was then reduced to 25°C, and protein expression was induced by the addition of IPTG to a final concentration of 0.2 mM. The culture was incubated overnight at 25°C with shaking. Cells were harvested by centrifugation at 5000 rpm for 15 min.

### 4.2 Purification of wtIGF‐II and mutIGF‐II

The cell pellet was suspended in lysis buffer (20 mM Tris‐HCl, pH 8.0, 300 mM NaCl, 10 mM imidazole, 6 M Guanidinium chloride, and 15 mM 2‐mercaptoethanol) and lysed by sonication. The lysate was cleared by centrifugation at 18000 rpm for 30 min at 4°C. The supernatant was loaded onto Ni‐NTA column and washed with buffer of 20 mM Tris, pH 8.0, 300 mM NaCl, 10 mM imidazole, then eluted with a buffer of 20 mM Tris, pH 8.0, 300 mM NaCl, 500 mM imidazole. The eluate from Ni‐NTA column was diluted 10‐fold by volume with refolding buffer (5 mM glycine, 0.5 mM Arginine, and 5 mM cysteine, pH 10.0) and incubated at 4°C for 48 h to promote the refolding.

### 4.3 Surface Plasmon Resonance (SPR) Assay

Surface plasmon resonance (SPR) data were generated using a Biacore X100 at 25°C using HBS‐EP+ as running buffer. Streptavidin was immobilized on a CM5 sensor chip using standard amine coupling chemistry. Biotinylated wtIGF‐II, mutIGF‐II, or M6Pn ligands were captured on the streptavidin‐functionalized sensor chip. Single‐cycle kinetics assays were conducted by injecting increasing concentrations of the recombinant extracellular domains of human IGF‐IIR, IGF‐IR, and IR‐A. Data were analyzed using the evaluation software.

### 4.4 Cell Culture

HepG2 and Huh7 cells were cultured in low‐glucose DMEM medium supplemented with 10% fetal bovine serum (FBS), 1% penicillin‐streptomycin, 1% non‐essential amino acid (NEAA), 1% sodium pyruvate, and 1% L‐glutamine. MDA‐MB‐231, U87, and SK‐BR‐3 were cultured in low‐glucose DMEM medium supplemented with 10% FBS and 1% penicillin‐streptomycin. All cell lines were grown at 37°C in a humidified 5% CO_2_ atmosphere.

### 4.5 Detection of pAKT Levels in IGF‐IR‐ or IR‐A–Overexpressing HepG2 Cells

HepG2 cells were seeded in 6‐well plates and cultured overnight. The next day, cells were transfected with 2 µg of IGF‐IR or IR‐A expression plasmid per well and incubated for 24 h. The medium was then replaced with fresh Opti‐MEM, and cells were cultured for an additional 24 h. Transfected HepG2 cells were harvested, counted, and reseeded into new 24‐well plates. After attachment, cells were treated with 20 nM wtIGF‐II or mutIGF‐II for 15 min, followed by lysis and analysis of pAKT levels by Western blot.

### 4.6 Cellular Internalization and Depletion of NA‐650

HepG2 cells were seeded into 48‐well plate. After overnight culturing, cells were treated with NA‐650 and biotinylated wtIGF‐II or mutIGF‐II for the indicated time points. After treatment, cells were harvested and lysed for in‐gel fluorescence analysis. For the depletion assay, 30 µL culture medium was collected for in‐gel fluorescence analysis.

### 4.7 Development of Degraders of EGFR and PD‐L1

Antibody (2 mg/mL) was reacted with 25 molar equivalents of NHS‐PEG_12_‐DBCO in PBS at room temperature (RT) overnight. Following purification with 50 kDa Amicon centrifuge filter units, the concentration of DBCO‐labeled antibody was tested using a BCA assay. Separately, wtIGF‐II or mutIGF‐II (2 mg/mL) was reacted with 8 molar equivalents of NHS‐PEG_12_‐azide in sodium carbonate buffer (pH 11.0) at RT for 10 min. Excess NHS‐PEG_12_‐azide was removed by 3 kDa Amicon centrifuge filter units. The purified‐DBCO labeled antibody (1 mg/mL) was then incubated with 10 molar equivalents of azide‐modified wtIGF‐II or mutIGF‐II at 4°C for 72 h. The average number of wtIGF‐II or mutIGF‐II molecules conjugated to each antibody was determined by MALDI‐TOF MS.

### 4.8 Characterization of NHS‐PEG_12_‐azide Labeling Site on mutIGF‐II

A 10 ng of each analyte was injected respectively into a Waters ACQUITY UPLC M‐Class system coupled to a timsTOF fleX mass spectrometer (Bruker Scientific, LLC, Bremen, Germany). The constantly isocratic elution flow rate was set at 3 µL/min for direct infusion with 80% mobile phase A (optimal water with 0.1% formic acid) and 20% mobile phase B (acetonitrile with 0.1% formic acid). The timsTOF fleX was calibrated according to the manufacturer's guidelines. The source parameters were set as follows: capillary voltage 1500 V, dry gas 3.0 L/min, and dry temp 180°C. The temperature of the ion transfer capillary was set to 180 °C. The full‐MS scan was set at *m/z* 150–2200 in positive ion mode. The MRM method was used to select precursor ions for fragmentation with an isolation window of 2 Da. The collision cell RF was set at 1500.0 Vpp, where the collision energy values were set at 40 and 60 eV. Bruker DataAnalysis 6.1 was utilized to deconvolute the monoisotopic mass and generate the mass spectra. The fragment ion elucidations were conducted via MS‐Product within Protein Prospector (v.6.6.4, University of California, San Francisco).

### 4.9 MALDI‐TOF MS

Samples were characterized by the following method: Matrix solution was made by dissolving sinapinic acid (SA) in 70% Acetonitrile/H_2_O with 0.1% trifluoroacetic acid (TFA) at a final concentration of 20 mg/mL. The sample was absorbed on Omix C4 pipette tips, followed by washing with 0.1% TFA five times and then eluted with 15 µL 75% Acetonitrile/H_2_O. After desalting the sample with Omix C4 pipette tips, 1 µL sample solution and 1 µL SA solution were spotted on the MALDI target plate and mixed thoroughly before the spot was allowed to dry under room temperature. MALDI‐MS spectra were acquired on a Bruker RapifleX MALDI TOF mass spectrometer (Bruker Scientific, LLC, Bremen, Germany) operated in linear positive ion mode. The smartbeam laser was set to 100% with a few thousand shots (labeled on each of the spectrum) per spot at a repetitive rate of 1000 Hz, and detector gain was set at 600 V for the experiments after method optimization. Spectra were processed in Bruker flexAnalysis 4.2.

### 4.10 Degradation Assays for EGFR and PD‐L1

Cells were seeded into 24‐well plates and incubated overnight. The following day, cells were treated with the indicated degraders for 24 h or 48 h prior to collection for Western‐blot analysis.

### 4.11 Generation of IGF‐IIR Knockdown Huh7 Cells

Huh7 cells were seeded into 24 well‐plate and incubated overnight. Cells were transfected with an sgRNA plasmid (Horizon Discovery) using FuGENE reagent in Opti‐MEM media for 48 h. Following transfection, the medium was replaced with fresh medium containing 2 µg/mL puromycin for selection. The selection medium was refreshed every other day for 2 weeks. Surviving cells were trypsinized and seeded at low density in 100 mm plate containing medium with 1 µg/mL puromycin to isolate single‐cell clones. Individual clones were picked, trypsinized, and expanded into a 6‐well plate in maintenance medium with 1µg/mL puromycin. Upon reaching 80% confluency, the cells were trypsinized and transferred to 100mm plate for further expansion.

### 4.12 Flow Cytometry

Cells were seeded into 12‐well plates and cultured overnight. Harvested cells were detached using trypsin‐EDTA and washed three times with PBS, followed by incubation with an anti‐IGF‐IIR antibody (ab124767, Abcam) for 30 min on ice. Cells were washed three times with PBS prior to incubation with an Alexa Fluor 647‐conjugated anti‐rabbit secondary antibody (A‐21244, Thermo Fisher Scientific). Following three additional washes with PBS, cells were resuspended in 500 µL of PBS containing 1% BSA and DAPI. Samples were analyzed using a Thermo Fisher Attune NxT flow cytometer. The data was analyzed with the FlowJo software.

### 4.13 Western Blot

Following three washes with PBS, cells were lysed by 1x RIPA buffer (25 mM Tris, pH 7−8, 150 mM NaCl, 0.1% (w/v) sodium dodecyl sulfate (SDS), 0.5% sodium deoxycholate, 1% (v/v) Triton X‐100) with protease inhibitors. After 15 min incubation on ice, cells were harvested into 1.5 mL tubes and centrifuged for 15 min at 16000 g. The supernatant was transferred into a new tube, and protein concentrations were determined using a BCA assay. The total protein amount and volume of each sample were adjusted to be the same by adding ddH_2_O. 4x Laemmli loading dye was added to each sample, followed by boiling at 99°C for 5 min. Boiled samples were loaded on to 7.5% or 12.5% SDS‐PAGE and transferred to PVDF membrane. Membranes were blocked with 5% (w/v) non‐fat dry milk for 1 h at room temperature (RT), followed by overnight incubation with primary antibody at 4°C overnight. The membrane was washed with TBST for three times and incubated with HRP‐conjugated secondary antibody and RT for 1 h. Following three additional washes with TBST, protein bands were visualized using Clarity ECL Substrate and imaged using a ChemiDoc MP imaging system. Band intensities were quantified using ImageJ software.

### 4.14 mutIGF‐II Based Fusion Protein Degrader Plasmid Construction

Coding sequences for mutIGF‐II (Del1‐7, Y27L) containing an N‐terminal FLAG tag were synthesized by Gene Universal, and the mammalian expression vector pCMV3 was purchased from Sino Biological. Plasmids were constructed using standard restriction enzyme cloning techniques. Briefly, PCR‐amplified IGF‐II inserts and pCMV3 vector were digested overnight with *Pac*I and *Xba*I at 37°C. Gel extracted digestion products were ligated with T4 DNA Ligase overnight at 16°C, and ligation products were ethanol precipitated, then resuspended in ddH_2_O. Purified ligation products were transformed into DH5α *E. coli* competent cells (Thermo Fisher) using the heat shock method before plated onto LB‐agar containing 50 µg/mL kanamycin and grown overnight at 37 °C. Colonies were screened by colony PCR, and positive clones were submitted for sequencing through Functional Biosciences. Next, sequence confirmed plasmid was utilized for the insertion of DNA segments encoding either rigid or flexible peptide linkers directly downstream of mutIGF‐II at the *Xba*I and *Asc*I restriction sites of the vector backbone. Lastly, the coding sequence for PD‐L1 targeting scFv was inserted directly downstream of the linker region at the *Asc*I and *Apa*I sites of the vector. Ultimately, a plasmid was constructed that encoded fusion proteins containing, from N to C terminus, an N‐FLAG mutIGF‐II, a flexible peptide linker, and a PD‐L1 target ligand scFv.

### 4.15 Expression and Secretion of Fusion Protein Degrader

Cells were seeded into 24‐well plate. After overnight incubation, cells were transfected with 200 ng plasmid using fuGENE as a transfection reagent (1:3 ratio) in Opti‐MEM. Twenty‐four h post‐transfection, the transfection medium was replaced with fresh DMEM, and the cells were incubated for an additional 48 h. Both conditioned medium and cell lysates were collected for Western blot analysis.

### 4.16 NA‐650 Internalization in 3D Tumor Spheroids

Huh7 cells were seeded at a density of 5,000 cells per well into ultra‐low attachment 96‐well plates U‐bottom wells and cultured for 7 days, with medium replenishment every other day. Spheroids were then treated with 25 nM NA‐650 and 500 nM mutIGF‐II–biotin for 24 h. Hoechst 33342 was added during the last 3 h of incubation at 37°C. Following treatment, spheroids were imaged on a Leica SP8 STED 3X super‐resolution microscope equipped with a 10× objective and a 10× eyepiece. Images were processed and quantified using ImageJ and Leica software.

### 4.17 Statistical Analysis

All quantitative data are presented as mean ± standard deviation (SD). Statistical analysis was carried out using GraphPad Prism. Comparisons between two groups were evaluated using an unpaired two‐tailed *t*‐test. For comparisons involving more than two groups, one‐way ANOVA was used, followed by Dunnett's post hoc test. A *p*‐value < 0.05 was considered statistically significant.

## Funding

NIH R35GM148266 (WT), R01DK071801 (LL), R01AG052324 (LL), S10OD028473 (LL), and S10OD025084 (LL).

## Conflicts of Interest

The authors declare no conflict of interest.

## Supporting information




**Supporting File**: advs74052‐sup‐0001‐SuppMat.docx.

## Data Availability

The data that support the findings of this study are available in the supplementary material of this article.
