# Supplementary material for: Potent and Selective IGF‐IIR‐Recruiting Bifunctional Molecules for Targeted Lysosomal Degradation of Extracellular and Membrane Proteins
Source: Adv Sci (Weinh). 2026 Jan 28;13(30):e18793. doi: 10.1002/advs.202518793 (PMC13248831; doi:10.1002/advs.202518793)
Supplement: Supplementary file 1 — Supporting File: advs74052‐sup‐0001‐SuppMat.docx. [file ADVS-13-e18793-s001.docx]

Supporting Information

**Potent and Selective IGF-IIR-Recruiting Bifunctional Molecules for Targeted Lysosomal Degradation of Extracellular and Membrane Proteins**

*Yuan Zhao, Yaxian Liao, Pengyun Li, Regina Stasser de Gonzalez, Xuankun Chen, Nicholas S. Nieto, Florence M. Brunel, Nick Cox, Joseph Stock, Matthew McHenry, Guangsen Fu, Penghsuan Huang, Wenxin Wu, Deqin Cai, Lingjun Li, Alexander N. Zaykov* and Weiping Tang**

[a] Y. Zhao, R. Stasser de Gonzalez, X. Chen, Dr. N. S. Nieto, Dr. D. Cai, Prof. Dr. L. Li, Prof. Dr. W. Tang
Lachman Institute of Pharmaceutical Development, School of Pharmacy, University of Wisconsin-Madison, Madison, WI, 53705, USA
E-mail: weiping.tang@wisc.edu

[b] Dr. P. Li, Dr. F. M. Brunel, Dr. N. Cox, Dr. J. Stock, Dr. M. McHenry, Dr. G. Fu, Dr. A. N. Zaykov
Chemical Biology, Novo Nordisk US R&D, 33 Hayden Ave., Lexington, MA 02421
E-mail: azaykov@penguin.bio

[c] Y. Liao, P. Huang, W. Wu, Prof. Dr. L. Li, Prof. Dr. W. Tang
Department of Chemistry, University of Wisconsin-Madison, Madison, WI, 53706, USA

Present address: [b] Dr. A. N. Zaykov, Penguin Bio, Indianapolis, IN, 46202, USA; Dr. P. Li, Full-life technologies, Bloomington, IN, 47401, USA

**Table contents:**

**Figure S1.** SPR sensorgrams showing binding interactions of wtIGF-II with IGF-IIR, IR-A, and IGF-IR

**Figure S2.** SPR sensorgrams showing binding interactions of mutIGF-II with IGF-IIR, but not to IR-A, and IGF-IR

**Figure S3.** SPR sensorgrams showing inhibition of wtIGF-II binding to IGF-IIR by low concentrations of mutIGF-II

**Figure S4.** SPR sensorgrams showing inhibition of wtIGF-II binding to IR-A only by high concentrations of mutIGF-II

**Figure S5.** SPR sensorgrams showing no inhibition of wtIGF-II binding to IGF-IR even by high concentrations of mutIGF-II

**Figure S6.** SPR sensorgrams showing 5-fold molar excess of mutIGF-II can compete wtIGF-II binding to IGF-IIR, but not IR-A and IGF-IR

**Figure S7.** Expression levels of IGF-IIR, IGF-IR, and IR in HepG2, U87, Huh7 and MDA-MB-231

**Figure S8.** Biotinylated wtIGF-II induced internalization of NA-650

**Figure S9.** Native mass spectrometry-based characterizations of 10 ng of mutIGF-II and mutIGF-II-PEG_12_-azide conjugates

**Figure S10.** Native top-down mass spectrometry-based characterizations of 10 ng of mutIGF-II, and mutIGF-II-PEG_12_-azide conjugates

**Figure S11.** Native top-down mass spectrometry-based characterizations of mutIGF-II, and mutIGF-II-PEG_12_-azide conjugates

**Figure S12.** wtIGF-II-based LYTAC degrader induced degradation of EGFR and PD-L1

**Figure S13.** Structure of azide-modified M6Pn

**Figure S14.** SPR sensorgrams showing binding interactions of M6Pn with IGF-IIR, IR-A, and IGF-IR

**Figure S15.** MALDI-TOF MS characterization of wtIGF-II and mutIGF-II labeled with NHS-PEG_12_-azide

**Figure S16.** MALDI-TOF MS characterization of cetuximab labeled with wtIGF-II and mutIGF-II

**Figure S17.** MALDI-TOF MS characterization of atezolizumab labeled with wtIGF-II, mutIGF-II, and M6Pn

**Figure S18.** MALDI-TOF MS characterization of pertuzumab labeled with mutIGF-II


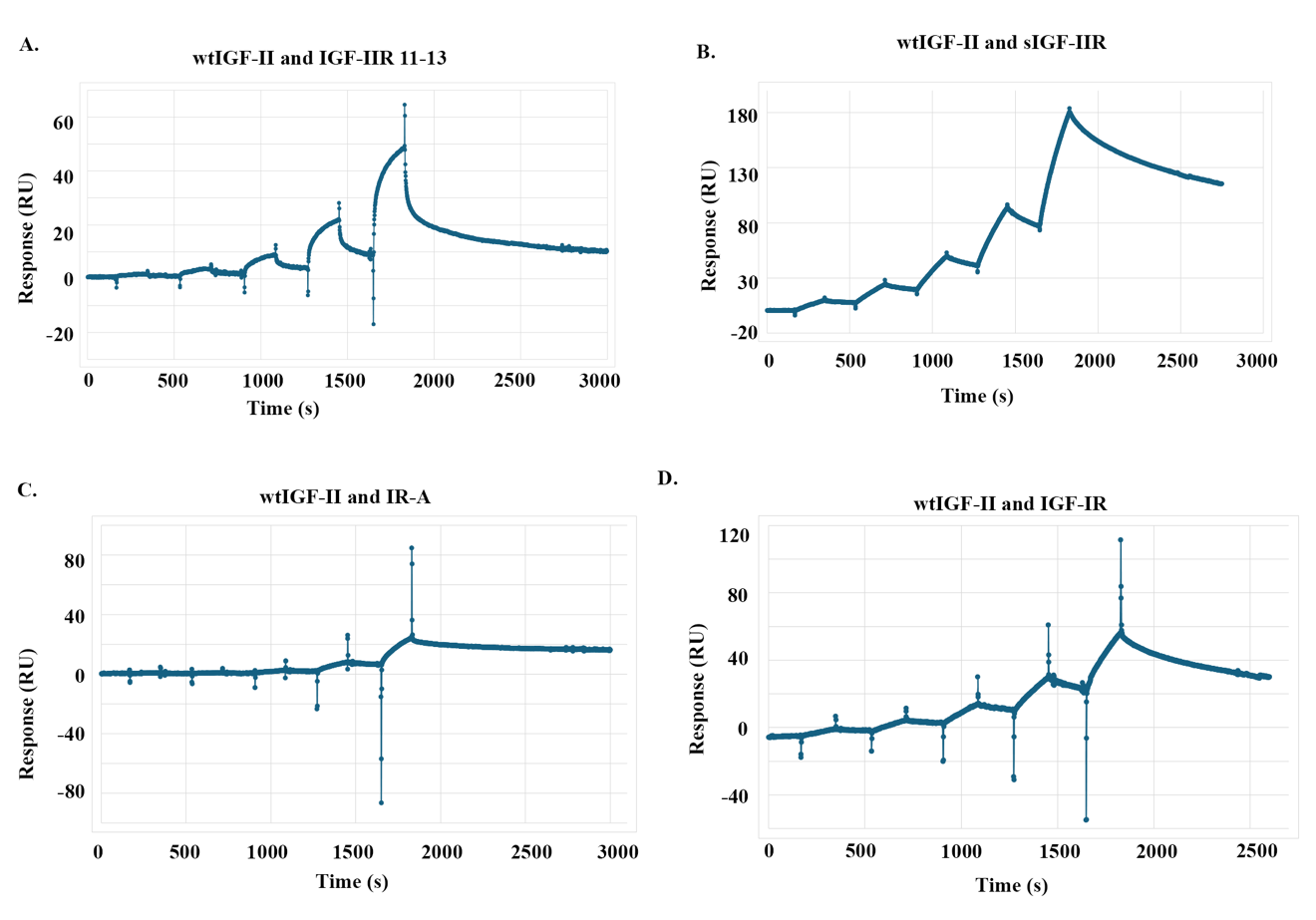
**Experimental section**

**Figure S1.** SPR sensorgrams showing binding interactions of wtIGF-II with IGF-IIR, IR-A, and IGF-IR. **(A)** Interaction between wtIGF-II and IGF-IIR extracellular domain 11-13 fragment. **(B)** Interaction between wtIGF-II and the full-length of IGF-IIR extracellular domain. **(C)** Interaction between wtIGF-II and recombinant IR-A. **(D)** Interaction between wtIGF-II and recombinant IGF-IR.


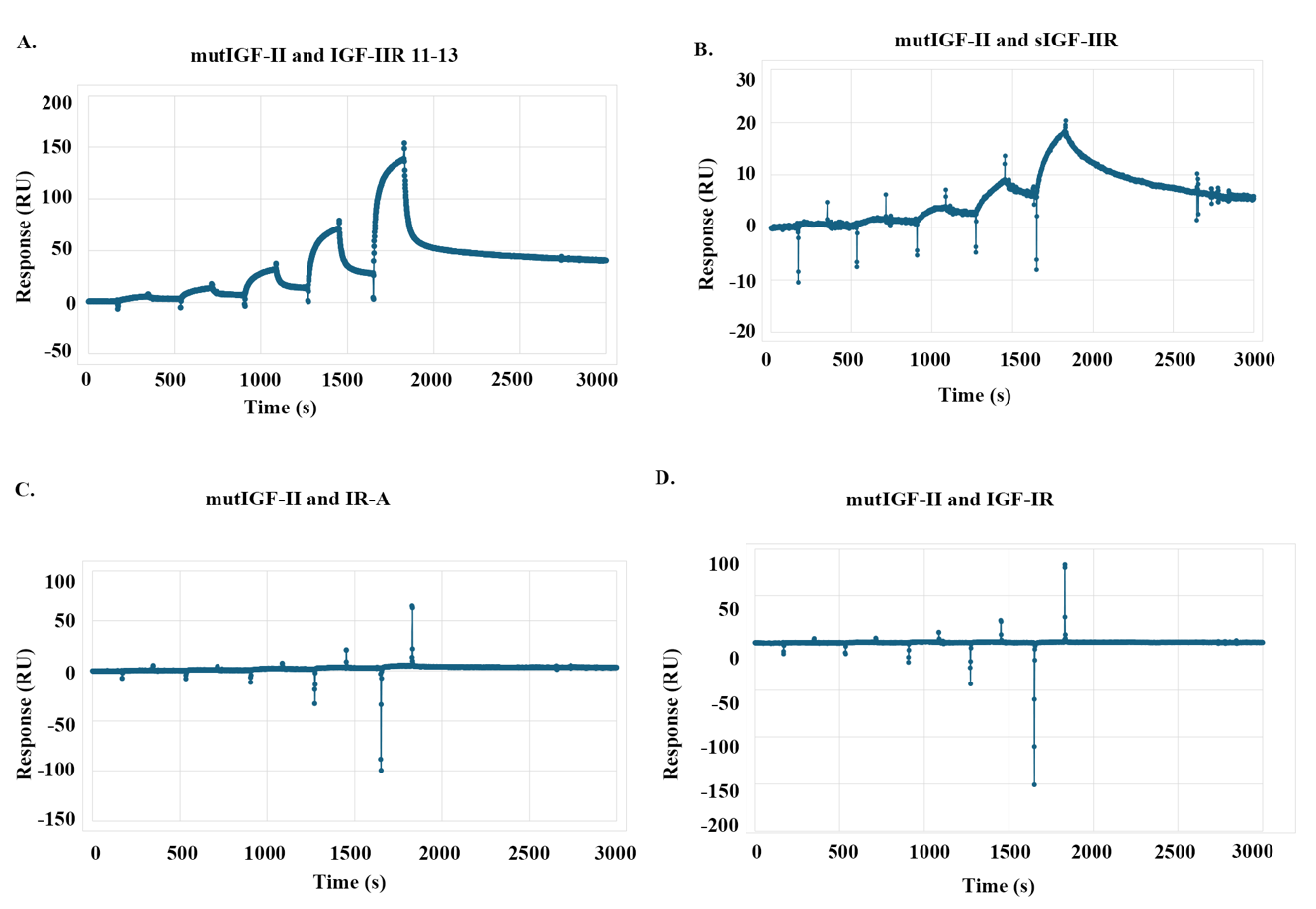


**Figure S2.** SPR sensorgrams showing binding interactions of mutIGF-II with IGF-IIR, but not to IR-A, and IGF-IR. **(A)** Interaction between mutIGF-II and IGF-IIR extracellular domain 11-13 fragment. **(B)** Interaction between mutIGF-II and the full-length of IGF-IIR extracellular domain. **(C)** Interaction between mutIGF-II and recombinant IR-A. **(D)** Interaction between mutIGF-II and recombinant IGF-IR.


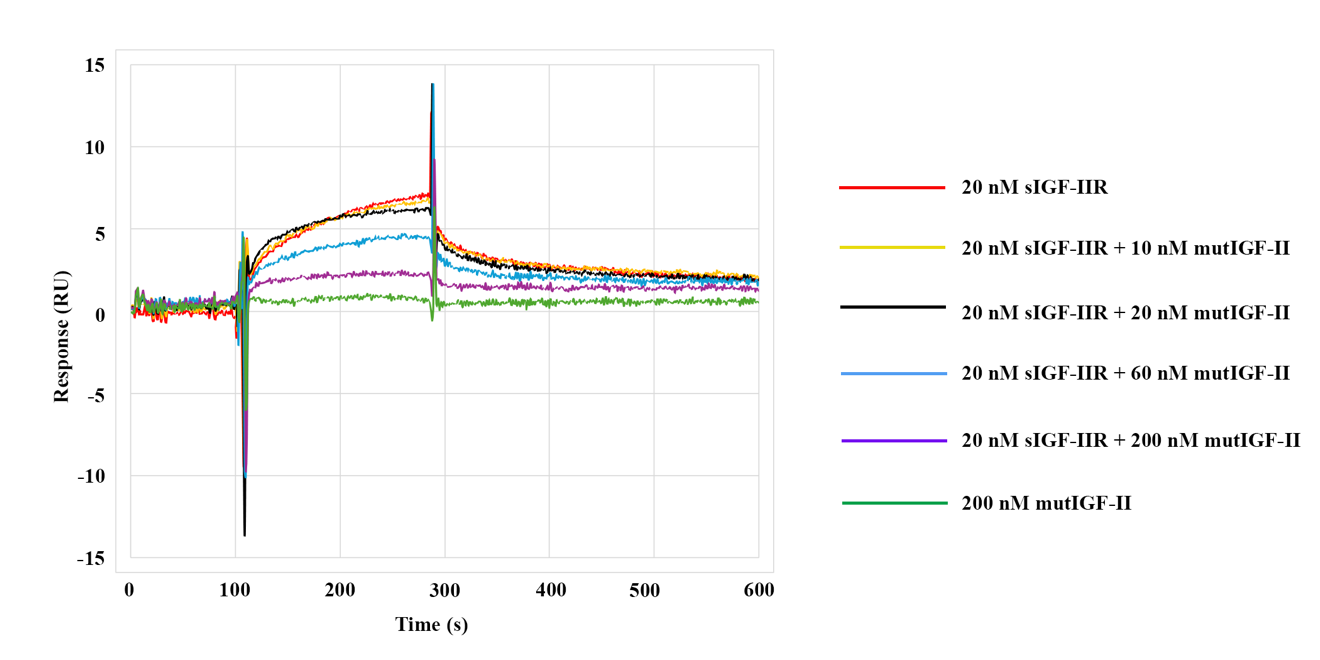


**Figure S3.** SPR sensorgrams showing inhibition of wtIGF-II binding to IGF-IIR by low concentrations of mutIGF-II.


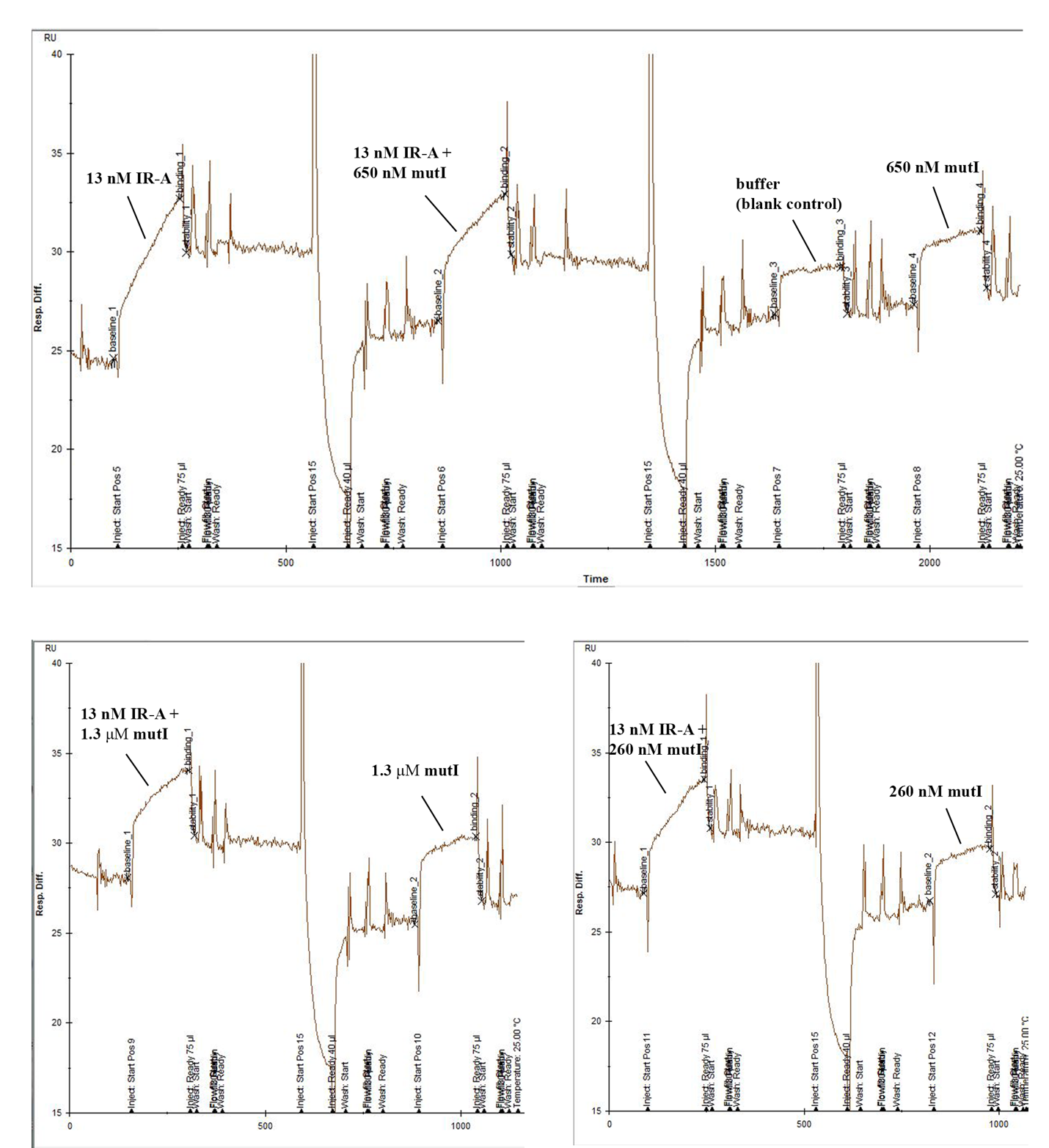


**Figure S4.** SPR sensorgrams showing inhibition of wtIGF-II binding to IR-A only by high concentrations of mutIGF-II.


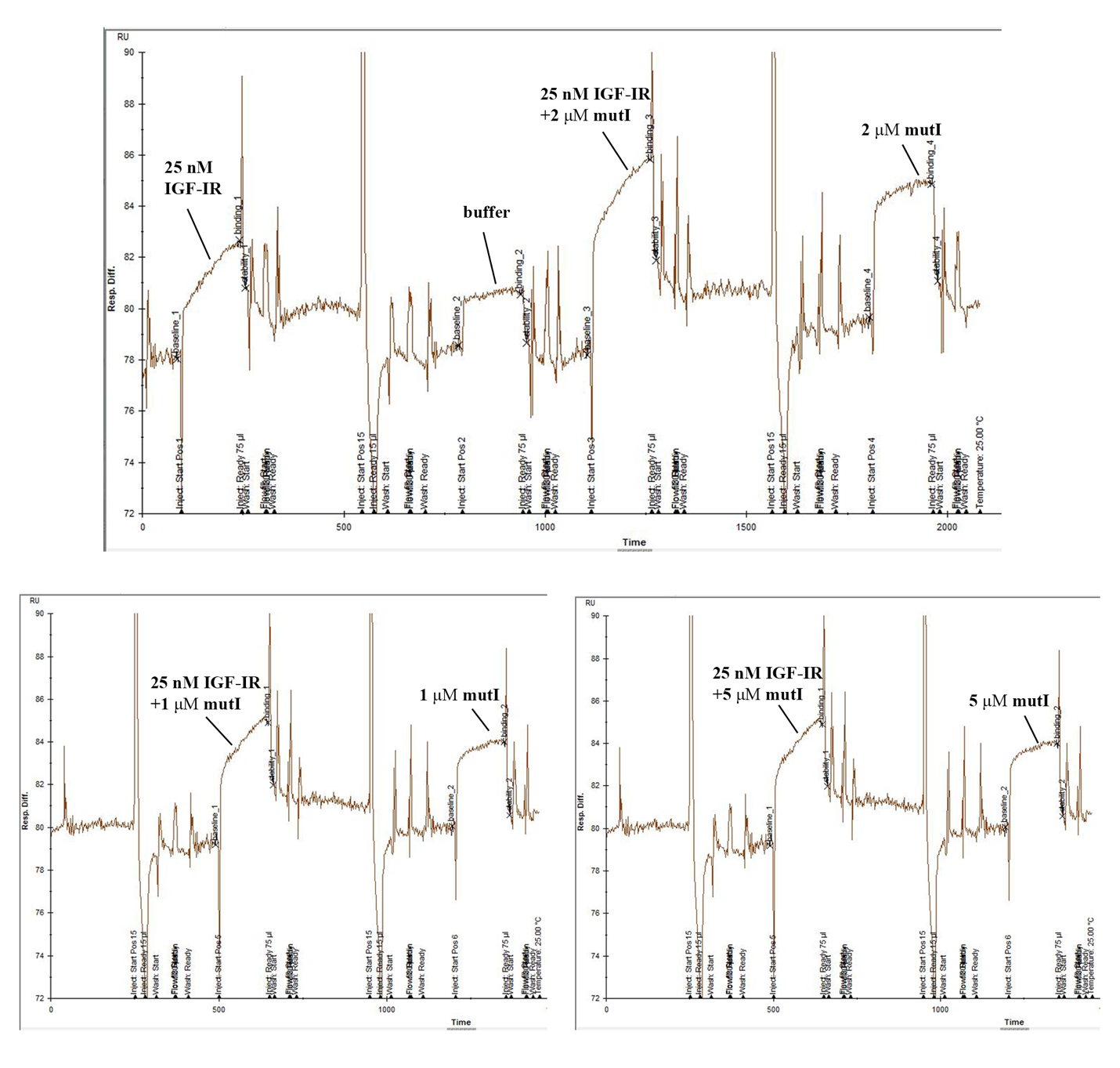


**Figure S5.** SPR sensorgrams showing no inhibition of wtIGF-II binding to IGF-IR even by high concentrations of mutIGF-II.


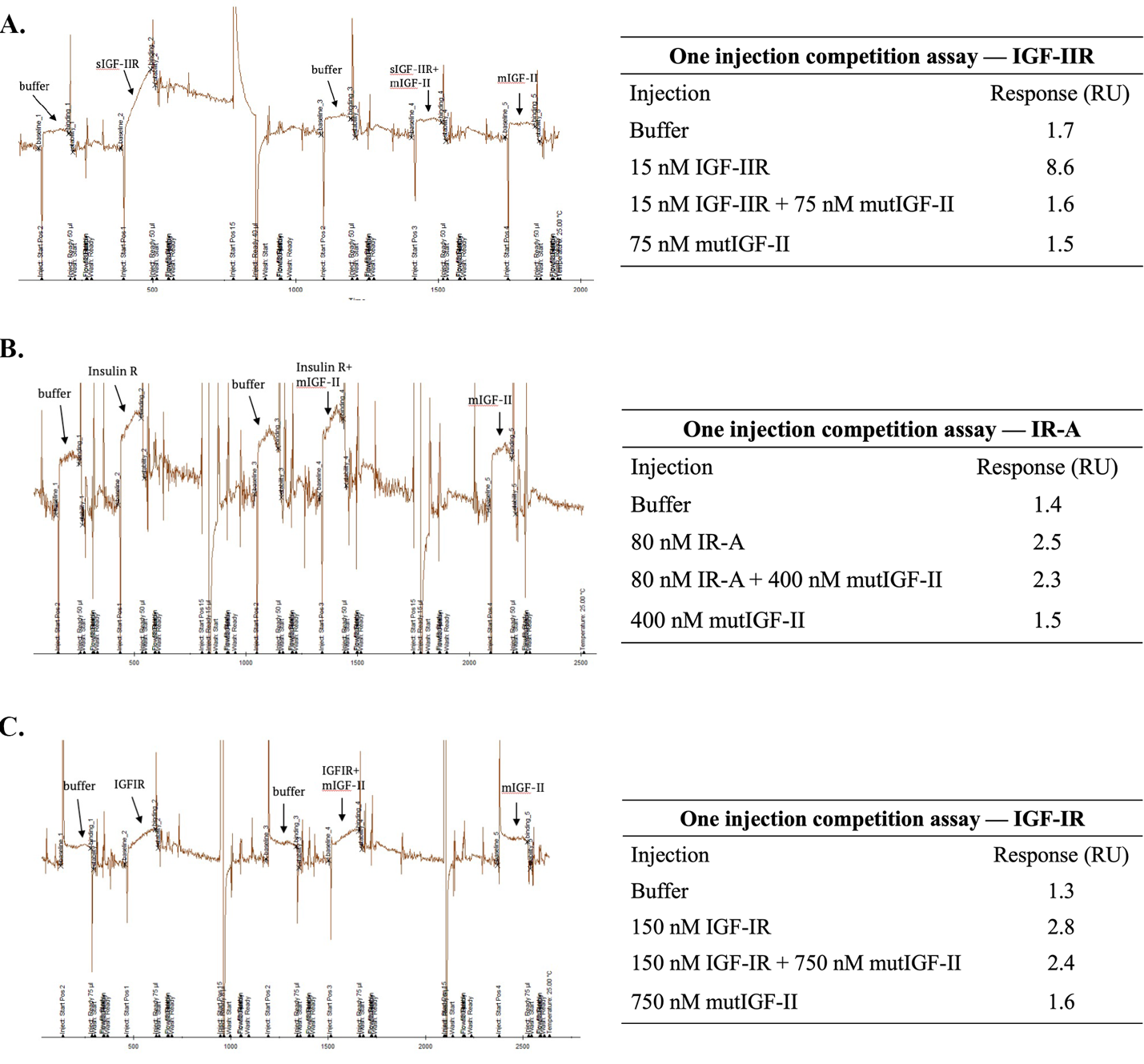


**Figure S6.** mutIGF-II can compete with wtIGF-IIR binding to IGF-IIR, but not IR-A and IGF-IR. **(A)** 5-fold molar excess of mutIGF-II inhibited binding between IGF-IIR and wtIGF-II. **(B)** 5-fold molar excess of mutIGF-II didn’t inhibit binding between IR-A and wtIGF-II. **(C)** 5-fold molar excess of mutIGF-II didn’t inhibit binding between IGF-IR and wtIGF-II.


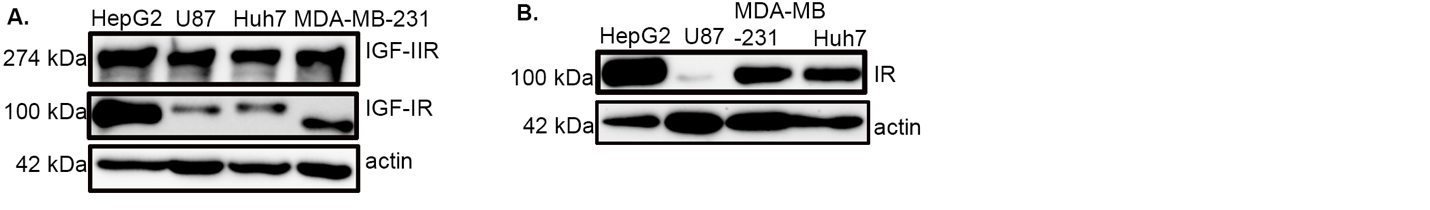


**Figure S7.** Expression level of IGF-IIR, IGF-IR, and IR in HepG2, U87, Huh7 and MDA-MB-231.


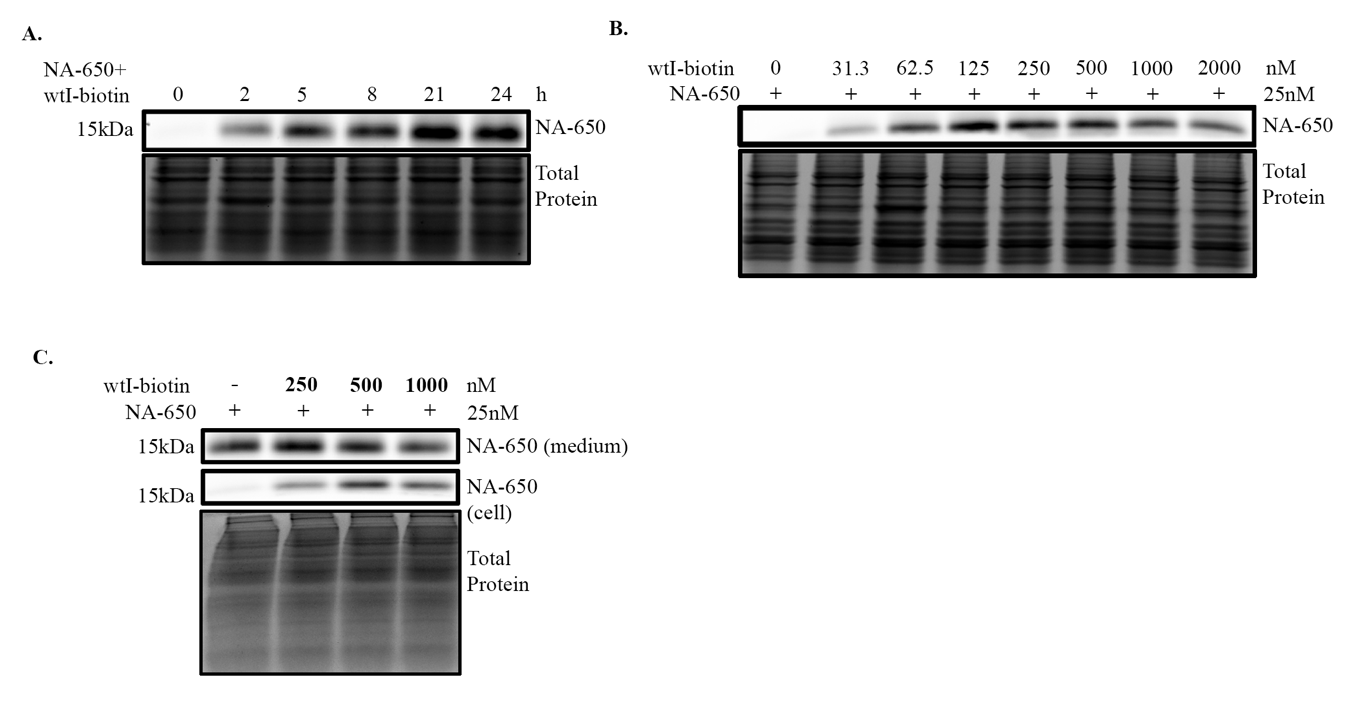


**Figure S8.** Biotinylated wtIGF-II induced internalization of NA-650. **(A)** Biotinylated wtIGF-II (500nM) induced NA-650 (25nM) uptake in a time-dependent manner. **(B)** Biotinylated wtIGF-II induced NA-650 uptake in a dose-dependent manner. **(C)** Biotinylated wtIGF-II induced NA-650 depletion from medium.


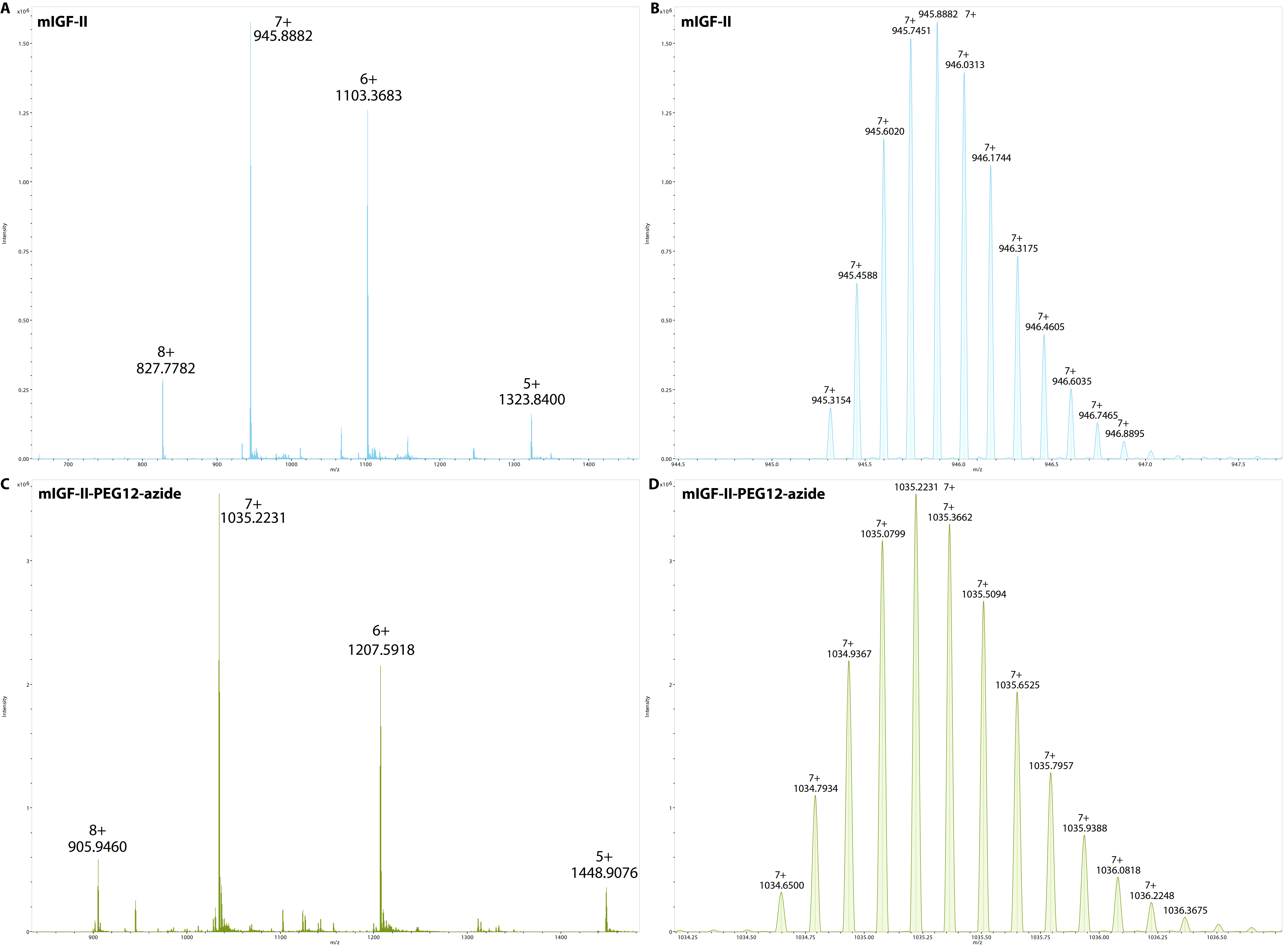


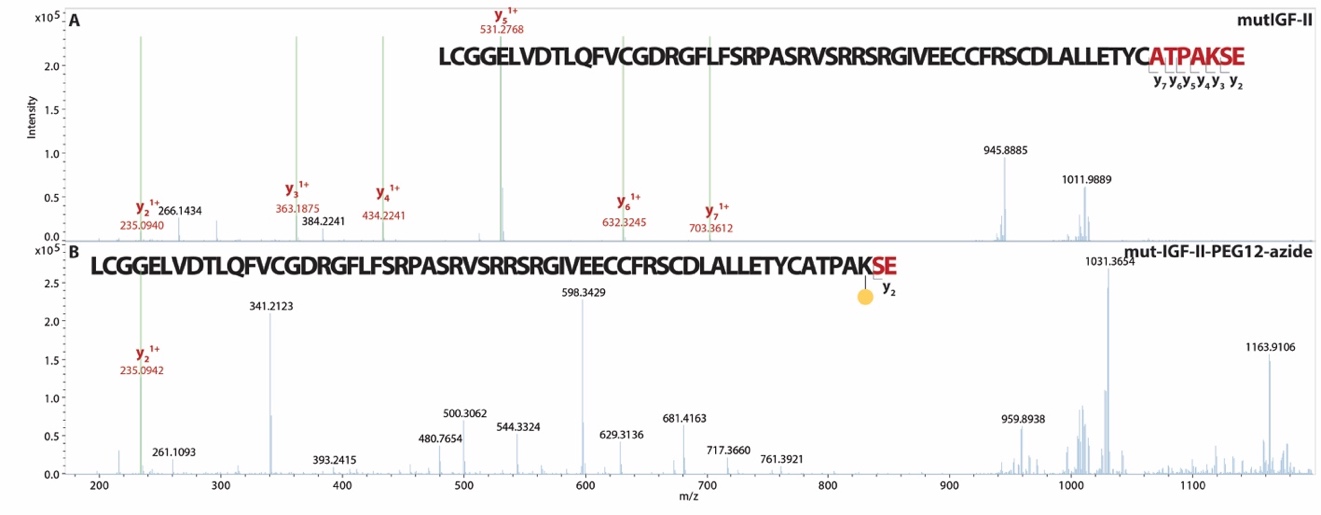
**Figure S9.** Native mass spectrometry-based characterizations of 10 ng of mutIGF-II and mutIGF-II-PEG_12_-azide conjugates. **(A)** Full-scan MS spectrum of mutIGF-II with charge states 5+ to 8+ detected, respectively. **(B)** Zoom-in of highest intensity charged analyte mutIGF-II (7+) showing the protein isotopic envelopes. **(C)** Full-scan MS spectrum of mutIGF-II-PEG_12_-azide conjugates with charge states 5+ to 8+ detected, respectively. **(D)** Zoom-in of highest intensity charged analyte mutIGF-II-PEG_12_-azide (7+) showing the protein isotopic envelopes.


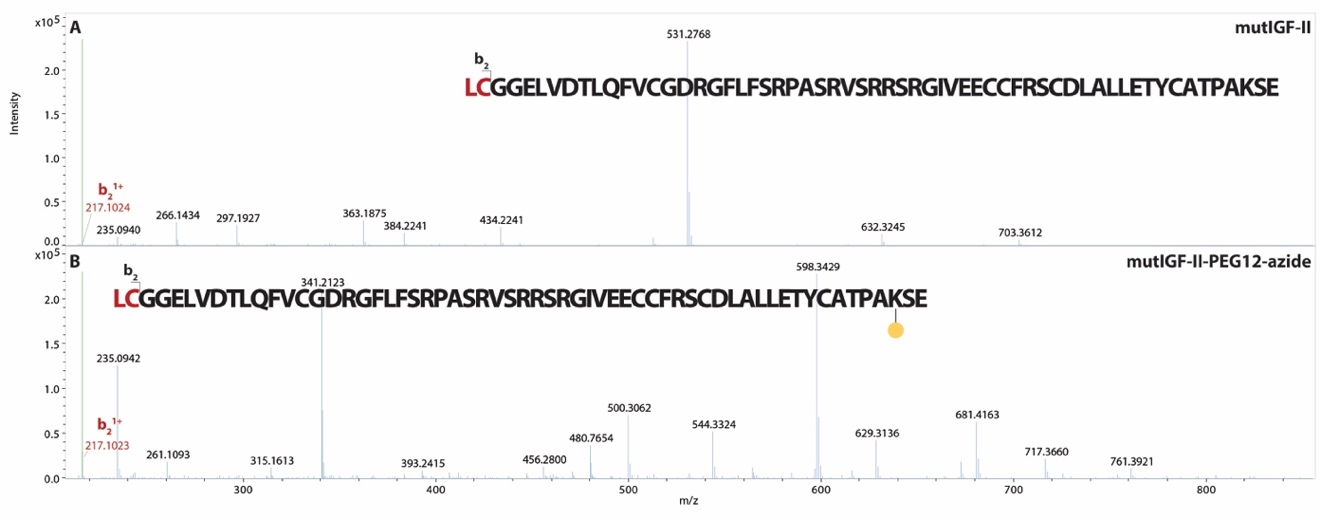
**Figure S10.** Native top-down mass spectrometry-based characterizations of 10 ng of mutIGF-II, and mutIGF-II-PEG_12_-azide conjugates. [M + 7H]^+^ ions of each of two analytes were isolated for MS/MS fragmentation, and related y-ions were elucidated on the lysine(K) residue at the C-terminal site of protein sequences. **(A)** The C-terminus sequence **ATPAKSE** of mutIGF-II was elucidated with y_2-7_^1+^ ions, which matched the fragmentation patterns of IGF. **(B)** The C-terminus sequence **ATPAKSE** of mutIGF-II-PEG_12_-azide was elucidated with only y_2_^1+^ ion, where the y_3-7_^1+^ ions cannot be detected after the PEG_12_-azide conjugated to the mutIGF-II.

**Figure S11.** Native top-down mass spectrometry-based characterizations of mutIGF-II, and mutIGF-II-PEG_12_-azide conjugates. [M + 7H]^+^ ions of each of two analytes were isolated for MS/MS fragmentation, and related b-ions were elucidated for the N-terminal site of protein sequences. **(A)** The N-terminus sequence LC of mutIGF-II was elucidated with b_2_^1+^ ions. **(B)** The N-terminus sequence LC of mutI-PEG_12_-azide was elucidated with b_2_^1+^ ions, where the mass shift of N-terminus fragments did not be observed after the modification with PEG_12_-azide conjugate.

**
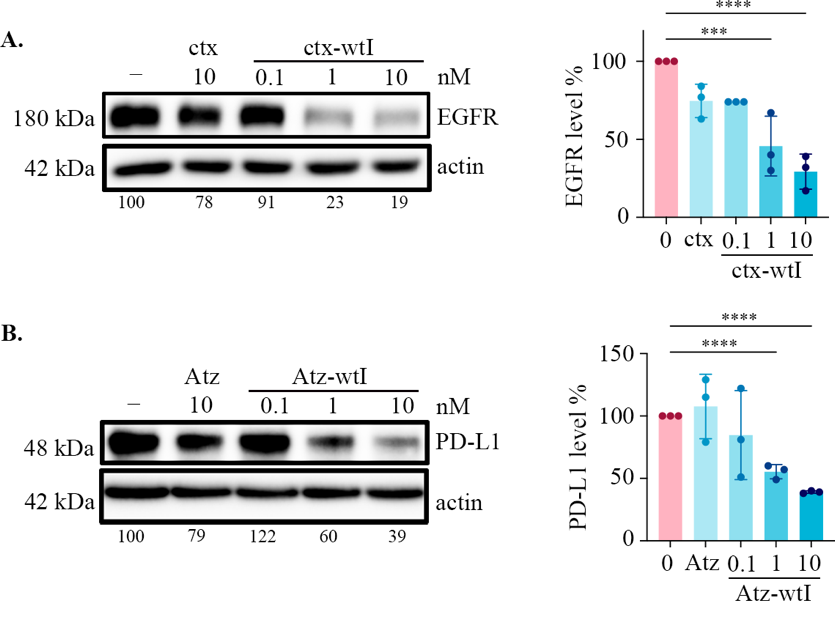
**

**Figure S12.** wtIGF-II based LYTAC degrader induced degradation of EGFR and PD-L1. **(A)** EGFR degradation induced by wtIGF-II based LYTAC degrader. **(B)** PD-L1 degradation induced by wtIGF-II based LYTAC degrader. Data are presented as mean ± SD. The statistical significance was assessed using one-way ANOVA, ****p* < 0.001, *****p* < 0.0001, ns: not significant.

**Figure S13.** Structure of azide modified M6Pn.


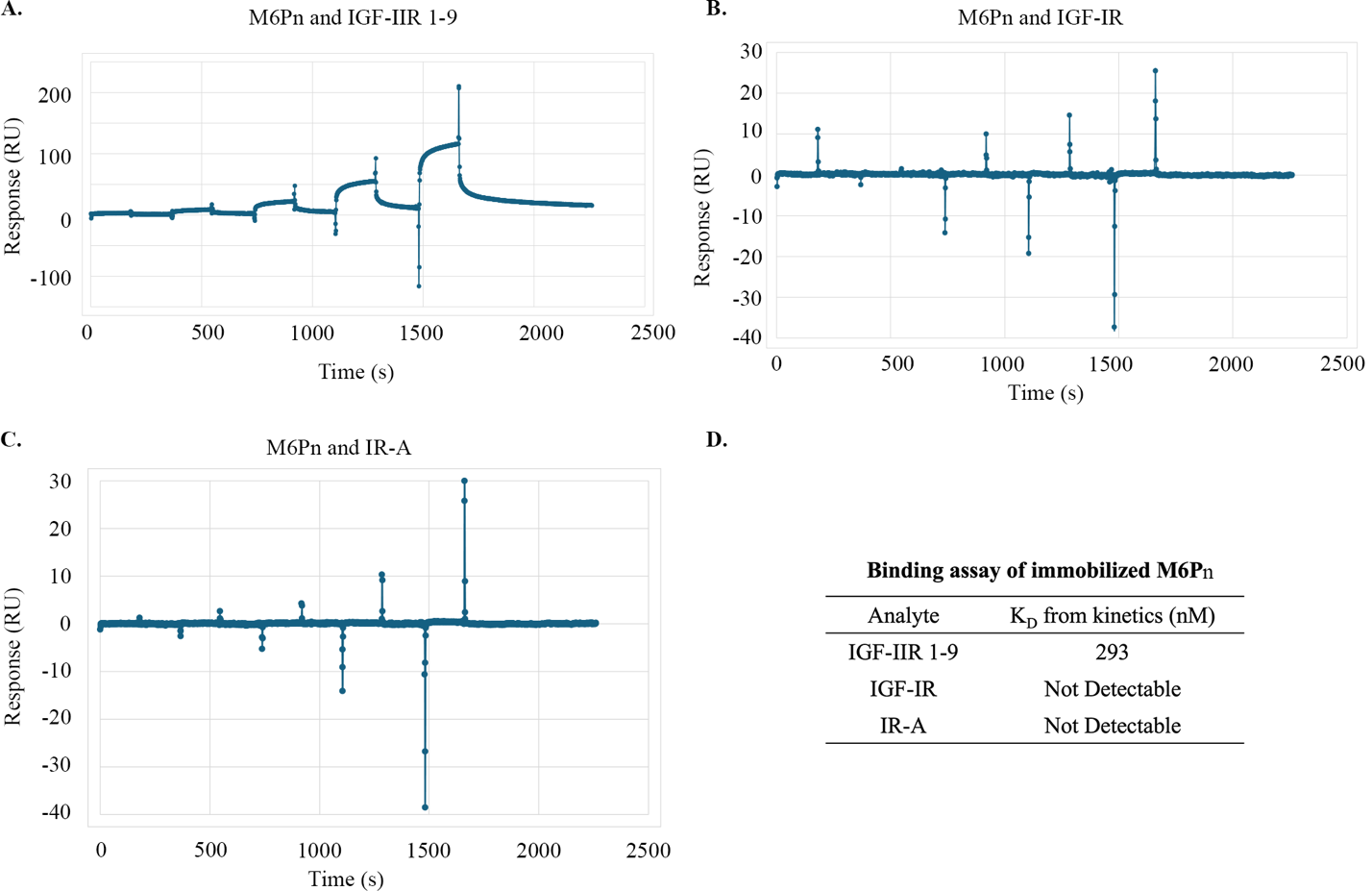


**Figure S14.** SPR sensorgrams showing binding interactions of M6Pn with IGF-IIR, IR-A, and IGF-IR. **(A)** Interaction between M6Pn and IGF-IIR extracellular domain 1-9 fragment. **(B)** Interaction between M6Pn and IGF-IR extracellular domain. **(C)** Interaction between M6Pn and recombinant IR-A.

**Figure S15.** MALDI-TOF MS characterization of wtIGF-II and mutIGF-II labeled with NHS-PEG_12_-azide


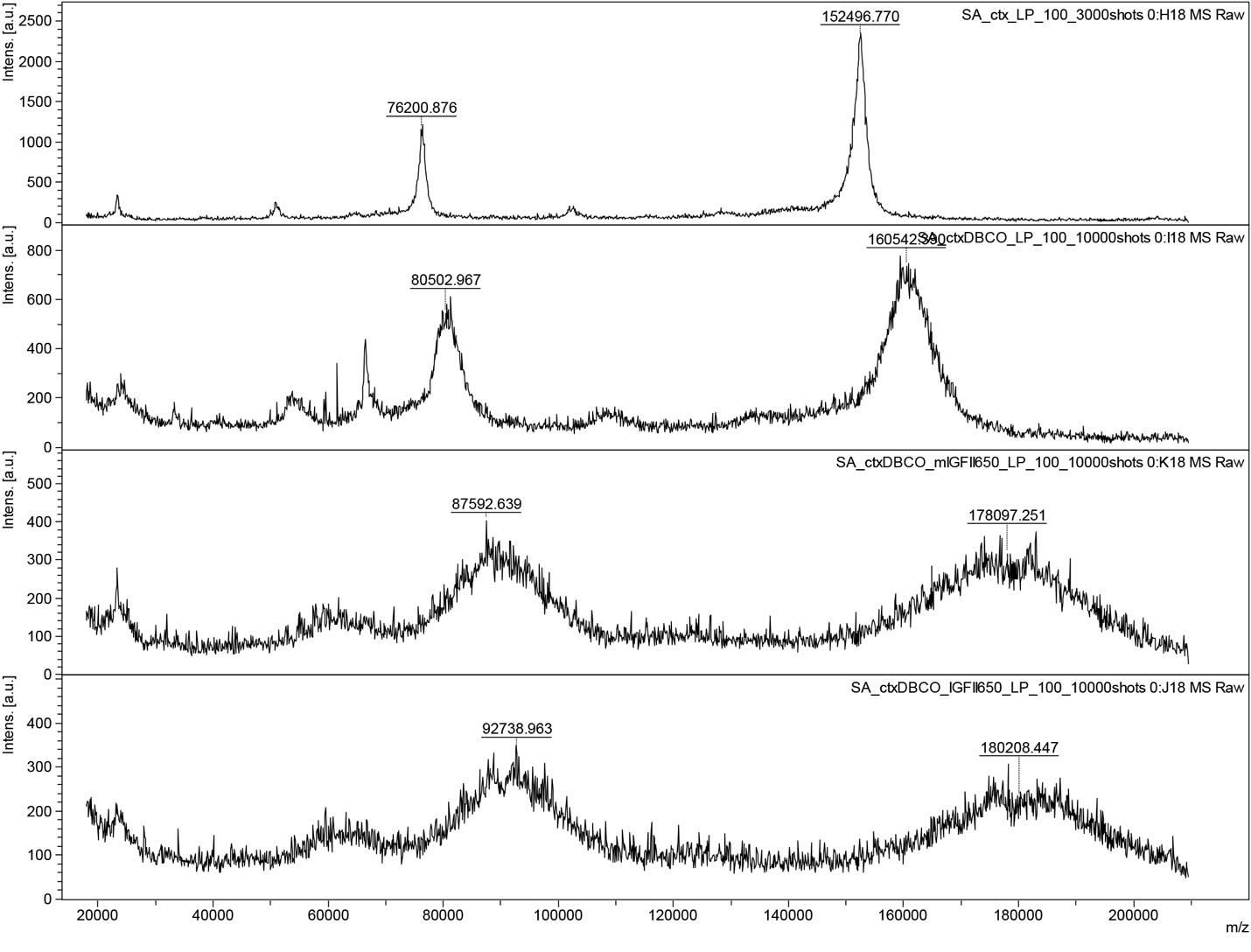


**Figure S16.** MALDI-TOF MS characterization of Cetuximab labeled with mutIGF-II and wtIGF-II.

**Figure S17.** MALDI-TOF MS characterization of atezolizumab labeled with wtIGF-II, mutIGF-II, and M6Pn


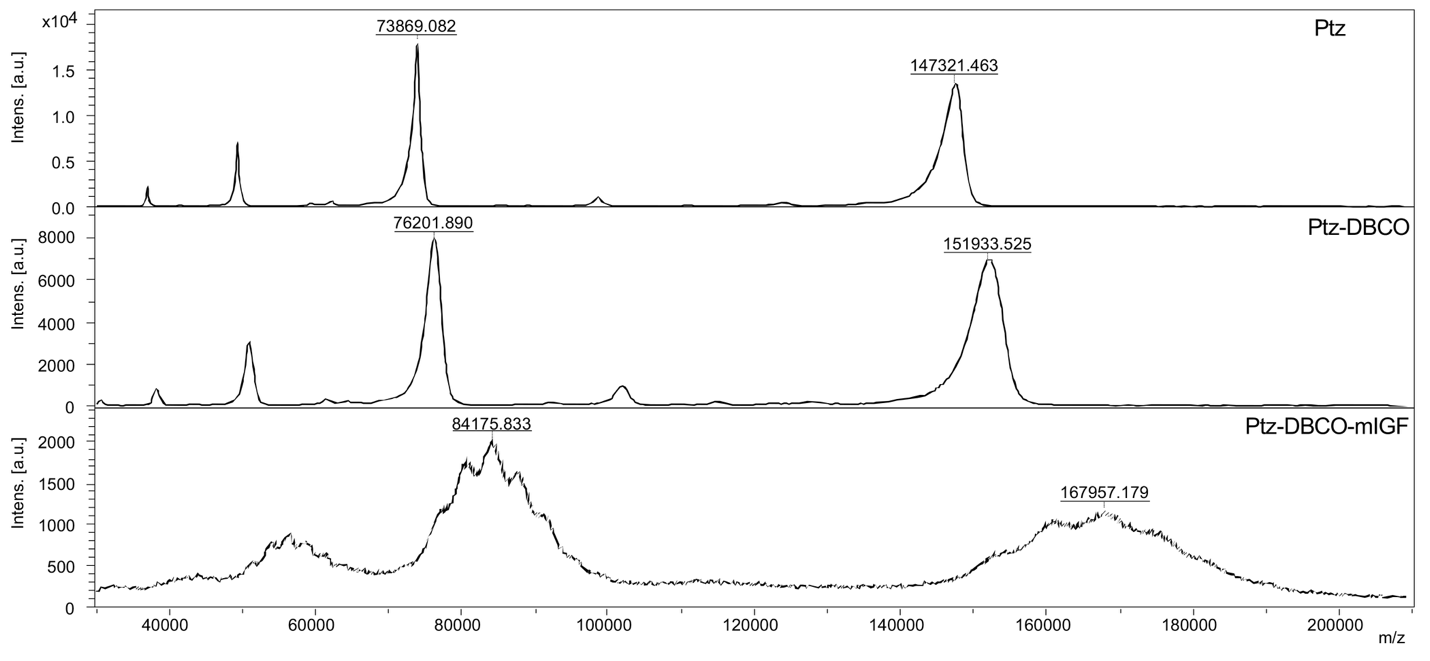


**Figure S18**. MALDI-TOF MS characterization of pertuzumab labeled with mutIGF-II
